# CAU-52: An Iron Metal–Organic Framework Containing Furandicarboxylate Linker Molecules

**DOI:** 10.1021/acs.inorgchem.5c00184

**Published:** 2025-04-07

**Authors:** Essam Alkhnaifes, Erik Svensson Grape, A. Ken Inge, Felix Steinke, Tobias A. Engesser, Norbert Stock

**Affiliations:** † Institut für Anorganische Chemie, Christian-Albrechts-Universität zu Kiel, Kiel 24098, Germany; ‡ Department of Chemistry, Stockholm University, Stockholm 10691, Sweden; § Department of Chemistry-Angström Laboratory; Synthetic Molecular Chemistry, Uppsala University, Uppsala 75120, Sweden

## Abstract

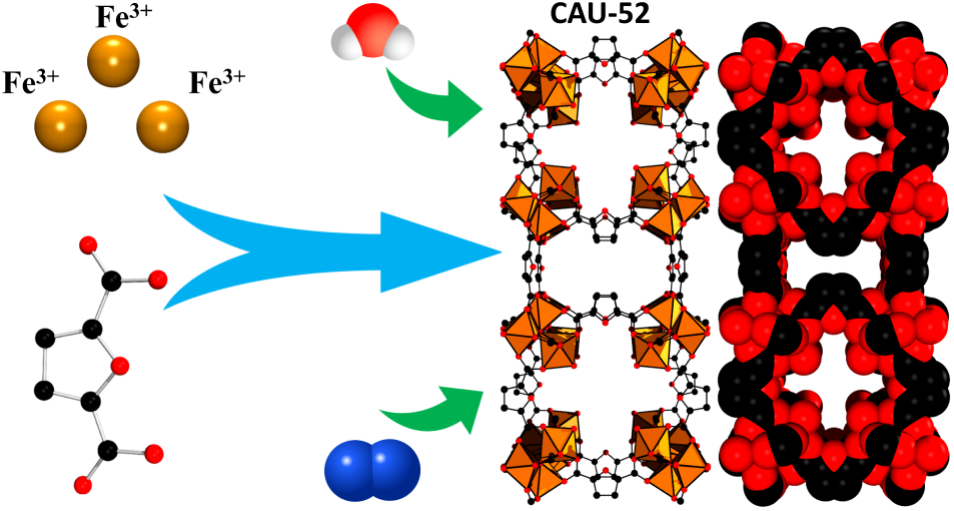

The V-shaped linker molecule 2,5-furandicarboxylic acid (H_2_FDC), which can be derived from lignocellulosic biomass, was used in a systematic screening with various iron salts and led to the discovery of a new iron-based metal–organic framework (Fe-MOF) with the composition [Fe_3_(μ_3_-O)(FDC)_3_(OH)(H_2_O)_2_]·5H_2_O·H_2_FDC, designated as CAU-52 (CAU = Christian-Albrechts-Universität zu Kiel). The crystal structure of CAU-52 was determined using 3D electron diffraction (3D ED) and further refined by Rietveld refinement against powder X-ray diffraction (PXRD) data. CAU-52 contains the well-known trinuclear [Fe_3_(μ_3_-O)]^7+^ cluster as the inorganic building unit (IBU) that is six-connected by FDC^2–^ ions to form the **pcu** net. The connectivity leads to two types of cubic cages, similar to the ones observed in soc-MOFs. Comprehensive characterization of the title compound, including N_2_ and water vapor sorption measurements, confirmed its chemical composition. CAU-52 exhibits microporosity toward nitrogen with a type-I isotherm (77 K), yielding a specific surface area of *a*_s,BET_ = 1077 m^2^/g. The H_2_O sorption measurement at 298 K leads to an isotherm that exhibits three steps. The water sorption capacity was determined to be 390 mg/g, and it decreases slightly in subsequent sorption cycles. The MOF is stable up to 250 °C in air and chemically resistant in various solvents.

## Abstract 2

The use of a V-shaped linker resulted in the formation of a MOF with **pcu** topology. Structure determination was accomplished by 3D electron diffraction and Rietveld refinement. The microporous MOF exhibits high water uptake in sorption measurements at 298 K, which diminishes slightly in successive cycles.

## Introduction

Metal–organic frameworks (MOFs) containing iron in the inorganic building unit (IBU) have been the subject of extensive studies due to the high abundance,^1^ low toxicity, and biocompatibility of iron.^2,3^ Thus, Fe-MOFs have been reported as possible candidates for applications in the fields of, for example, water sorption,^4^ gas separation,^5,6^ drug delivery^7^ and catalysis.^8,9^

Usually, Fe-MOFs are obtained as crystalline products under solvothermal reaction conditions, and commonly used solvents are water,^10^*N*,*N*-dimethylformamide (DMF)^11^ and acetic acid.^12^ An efficient methodology to screen a wide range of reaction conditions for MOF syntheses is high-throughput (HT) methods. Compared to conventional methods, high-throughput methods can be used to carry out a large number of reactions in a short time using small amounts of chemicals.^11,13^ Thus, they allow a systematic and efficient study of complex parameter fields and provide a better understanding of the influence of chemical and process parameters on the resulting reaction product. HT methods are based on the concepts of parallelization, miniaturization, and automation in synthesis and characterization, which are implemented to varying degrees in the HT set-ups of different research groups and were recently also combined with AI (GPT-4).^14−16^ Recently, a video protocol demonstrating how high-throughput methods are employed in our group has been published, and technical drawings of the reactors have also been provided.^17^

The choice of the linker is also important, as it determines the topology of the framework together with the IBU. While many MOFs have been prepared using highly symmetric linker molecules, such as terephthalic acid and its derivatives, recent investigations have explored the use of less symmetric linkers, using dicarboxylic acids with different geometries, such as isophthalic and phthalic acid.^18^ Especially interesting for industrial applications are linker molecules that can be derived from environmentally friendly and sustainable raw materials.^19,20^ Lignocellulosic biomass is such a low-cost, abundant resource whose potential for the production of sustainable chemicals has not yet been fully realized. The monosaccharides that can be derived from lignocellulose can be converted into valuable products, such as 5-hydroxymethylfurfural (HMF), which can then be further processed by selective oxidation to 2,5-furandicarboxylic acid (H_2_FDC).^21,22^ This linker has been used in the synthesis of Zn-, Fe-, In-, and Zr-MOFs,^23−26^ as well as Al-MIL-160.^27^ The latter MOF has been intensively studied for its excellent water sorption properties and is an example where the choice of linker molecules with hydrophilic groups is particularly important, as they can effectively shift the steep adsorption region to lower relative humidity values.^28^ Indeed, the combination of trivalent metals and aromatic, nonlinear (V-shaped) dicarboxylates has resulted in MOFs with high potential, for example, in atmospheric water harvesting. In addition to MIL-160,^27^ important examples include CAU-10,^29^ CAU-23,^30^ MOF-303, and MOF-333.^31^

In Fe-MOF chemistry, various di-, tri-, and tetracarboxylic acids have been employed. Notable examples containing trinuclear IBUs with the composition [Fe_3_(μ_3_-O)(RCO_2_)_6_]^+^^32,33^ include MIL-88,^34^ MIL-100,^3^ MIL-101,^11^ Fe-soc-MOF (PCN-250^12^/MIL-127)^35^ and PCN-333.^36^ These have been obtained as highly crystalline products under solvothermal reaction conditions. Nevertheless, there is a lack of information available on Fe-MOFs with V-shaped dicarboxylate linker molecules.^37,38^ Examples include Fe-MIL-59^10^/PCN-234^12^ with isophthalate ions, DNL-9(Fe)^39^ and PCN-233^12^ with 2,5-furandicarboxylate ions,^40^ which show promising sorption properties, e.g., toward water.^10^

Here, we report a systematic investigation of obtaining Fe-MOFs using the V-shaped linker 2,5-furandicarboxylate. Specifically, HT methods were used to study the system Fe^3+^/H_2_FDC/NaOH/water/cosolvent, using DMF and acetic acid as cosolvents, which resulted in the discovery of a new Fe-MOF with the framework composition [Fe_3_(μ_3_-O)(FDC)_3_(OH)(H_2_O)_2_]·5H_2_O·H_2_FDC (CAU-52). The structural determination from electron diffraction data, the chemical and thermal stability, and the sorption properties are reported.

## Experimental Section

### Materials

All reagents and solvents are commercially available and were used without further purification. These include: FeCl_3_·6H_2_O (Merck, > 99%), Fe(NO_3_)_3_·9H_2_O (Merck, > 99%), Fe_2_(SO_4_)_3_ (Riedel-de Haën, reinst), 2,5-furandicarboxylic acid (H_2_FDC, abcr >98%), NaOH (Grüssing, 99%), CH_3_COOH (AcOH, VWR, 100%), NaO_2_CH (Sigma-Aldrich, >99%), and *N,N*-dimethylformamide (DMF, Grüssing GmbH, >99%).

### Methods

The syntheses were carried out in a Memmert UNB 500 oven with forced ventilation using a predefined temperature–time program and custom-made steel multiclaves with Teflon inserts (total volume of 2.5 mL).^13^ The initial characterization by powder X-ray diffraction (PXRD) was carried out using a STOE Stadi MP and a STOE Stadi P-Combi diffractometer, both equipped with a MYTHEN 1K detector and using monochromated Cu Kα1 radiation. The PXRD data for structure refinement were collected on a STOE Stadi MP instrument equipped with a MYTHEN 1K detector and using Cu Kα1 radiation. Three-dimensional electron diffraction (3D ED) data were collected with a JEOL JEM 2100 LaB_6_ microscope operating at 200 kV, equipped with a Timepix hybrid pixel detector (see Supporting Information for further details). The thermal properties of the sample were studied by thermogravimetric analysis (TGA) and variable-temperature PXRD (VT-PXRD). TGA was carried out using a Linseis STA PT 1600 (air flow = 6 L/h, heating rate = 8 K min^–1^). The VT-PXRD measurements were performed by using a STOE Stadi-P Combi powder diffractometer (Cu Kα1 radiation) equipped with a capillary furnace. For this purpose, the analyzed sample was transferred to a 0.5-mm quartz capillary, heated in defined temperature steps, and a PXRD pattern was recorded. IR spectra were measured by using a Bruker ALPHA-P ATR MIR spectrometer. Variable-temperature diffuse reflectance infrared Fourier transform spectroscopy (VT-DRIFTS) was carried out with a Praying Mantis Diffuse Reflection Accessory from Harrick Scientific Products in a Bruker Vertex 70 FT-IR spectrometer, using a broadband spectral range extension VERTEX FM for full, mid, and far IR in the range of 6.000–80 cm^–1^. ^1^H NMR spectra were measured using a Bruker DRX 500 spectrometer. After the compound was dissolved in 10% NaOD/D_2_O, the red-brown iron oxide/hydroxide precipitate was separated by centrifugation, and the clear solution was used for the measurement. The energy-dispersive X-ray (EDX) analyses were carried out by EDX measurements and conducted using a Phillips ESEM XL30, equipped with a Si (Li) detector model Sapphire, SUTW (super ultrathin window). Elemental analysis was performed with a vario MICRO cube elemental analyzer from Elemental Analysensysteme GmbH. Sorption measurements were performed using a BELSORP-max analyzer (BEL Japan Inc.). Before the sorption measurements, the samples were treated for 16 h under vacuum pressure (*p* < 10^–2^ mbar) at a temperature of 100 °C.

### Synthesis

Investigation of the system Fe^3+^/H_2_FDC/NaOH/H_2_O/cosolvent, with the cosolvents DMF and CH_3_COOH, was carried out in our custom-made high-throughput reactors. The molar ratio of Fe^3+^/H_2_FDC/NaOH was kept constant, and the influence of the Fe(III) salt, as well as the impact of acetic acid and DMF on the product formation, was studied. Syntheses were carried out in 2.5 mL Teflon inserts. The Teflon inserts were placed in the custom-made steel multiclave and heated to 120 °C within 1 h, the temperature was kept for 6 h and then cooled down within 3 h to room temperature. The exact amounts of the reactants and solvents are given in Table S1 and the PXRD patterns of the reaction products are shown in Figure S1.

### Optimized Synthesis Conditions of CAU-52as and CAU-52

The optimized synthesis is as follows: a 2.5 mL Teflon reactor was charged with 800 μL of a solution of sodium furandicarboxylate (0.4 mmol, Na_2_FDC, *c* = 0.5 mol/L) and acetic acid (480 μL, 8.4 mmol). The reactants were thoroughly mixed, and a freshly prepared aqueous solution of Fe(III) chloride (720 μL, *c*(Fe^3+^) = 0.5 mol/L, 0.36 mmol) was added, followed by the homogenization of the mixture. The Teflon insert was placed in the custom-made steel multiclave and heated to 120 °C within 1 h, the temperature was kept for 6 h and then cooled down within 3 h to room temperature. The pH value of the solution before and after the synthesis was pH = 2. The yellow product, designated CAU-52as (as: as-synthesized), was filtered off, washed twice with 1.5 mL of H_2_O and dried for 3 h at 70 °C.

To obtain larger quantities of CAU-52as, representing the as-synthesized product, for detailed characterization, the optimized synthesis was repeated in six 2.5 mL Teflon reactors in parallel using our multiclave. PXRD confirms the formation of CAU-52as, but in one sample, H_2_FDC is observed as the second crystalline phase (Figure S2). The six samples were combined to yield a total of 488 mg.

To purify the material and obtain the final product, denoted as CAU-52, approximately 300 mg of CAU-52as was stirred in 5 mL of DMF for 4 days at room temperature, then collected by centrifugation. This was followed by stirring in 5 mL of deionized water for 6 days (replacing it four times), during which a color change of CAU-52 from orange in DMF to yellow in water was observed. The resulting washed product, CAU-52, was isolated by centrifugation and dried at 70 °C for 6 h in air, yielding 195 mg of the product, calculated as [Fe_3_(μ_3_-O)(FDC)_3_(OH)(H_2_O)_2_]·5H_2_O·H_2_FDC.

The PXRD patterns of CAU-52 before and after purification are shown in Figure S3. The reflections of the linker molecules are no longer visible, and the reflections of CAU-52 exhibit larger full width at half maximum (FWHM) values, indicating a lower long-range order.

## Results and Discussion

The title compound, CAU-52as, is obtained under solvothermal reaction conditions in the system Fe^3+^/H_2_FDC/NaOH/H_2_O/CH_3_COOH. The absence of NaOH as a pH modulator or acetic acid as a cosolvent leads only to X-ray amorphous products (Figure S1). The as-synthesized compound is obtained using a molar ratio of Fe^3+^:H_2_FDC:NaOH:CH_3_COOH = 0.9:1:2:21 and contains recrystallized linker molecules that can be partially removed by treatment with DMF, which is subsequently removed by stirring the sample in water. This sample is denoted CAU-52 and has the composition [Fe_3_(μ_3_-O)(FDC)_3_(OH)(H_2_O)_2_]·5H_2_O·H_2_FDC, as confirmed by elemental analysis, thermogravimetric measurements, and NMR spectroscopy using a standard (see below). Thus, the washing procedure only leads to the dissolution of recrystallized linker molecules, while linker molecules trapped in the pores are not removed. The product is only obtained as a submicrocrystalline powder, not suitable for single-crystal X-ray diffraction. Since structure determination from powder X-ray diffraction data was unsuccessful, the crystal structure was determined using three-dimensional electron diffraction (3D ED) measurements.

### Structure Determination of CAU-52

**Figure 1:**
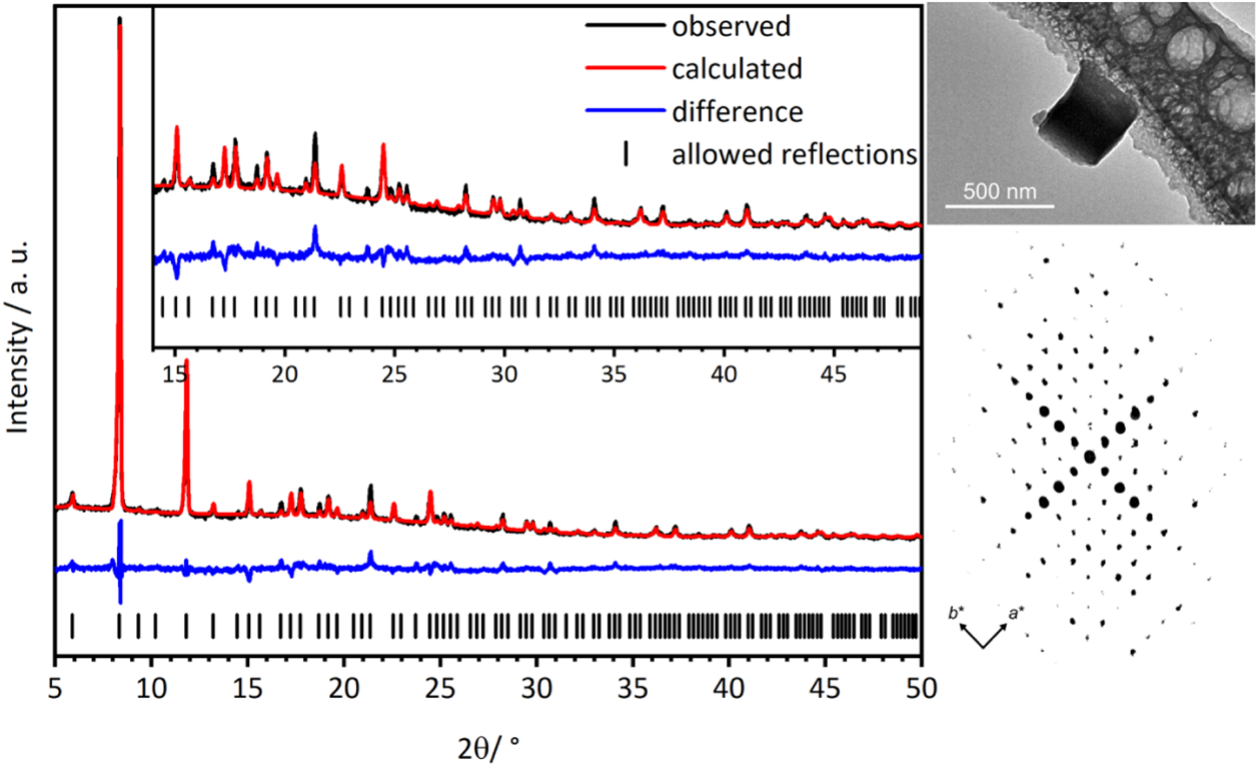
Left: final Rietveld plot of the structure refinement of [Fe_3_(μ_3_-O)(FDC)_3_(OH)(H_2_O)_2_]·5H_2_O·H_2_FDC, CAU-52. The observed curve is shown in black, the calculated curve in red, the difference curve in blue, and the positions of allowed reflections as black lines. Right: TEM-micrograph and reconstructed reciprocal space projection viewed along c* from electron diffraction (3D ED) of a single crystal of CAU-52as.

The crystal structure of CAU-52 was determined from 3D ED data, more specifically continuous rotation electron diffraction (cRED) data, collected on a small crystallite of CAU-52as (<0.5 μm, Figure 1). These data were collected using a JEOL JEM2100 TEM, equipped with a Timepix detector from Amsterdam Scientific Instruments, while continuously rotating the crystal at 0.45° s^–1^. The experiments were carried out using Instamatic,^41^ with data reduction performed through XDS.^42^ The acquired intensities were then used to solve the structures with SHELXT,^43^ and refined using SHELXL,^44^ with electron scattering factors extracted from SIR2014.^45^ The reconstructed reciprocal space projection viewed along c*, lattice parameters, and crystallographic data are presented in Figure S4 and Table S2.

The crystal structure of CAU-52as was employed as a starting model for structure refinement against PXRD data for the washed sample CAU-52 using the Rietveld method^46^ in Topas Academic.^47^ Details of the structure refinement are given in Section S2 and the final Rietveld plot is presented in Figure 1. The crystallographic data of the Rietveld refinement are presented in Table 1. The CCDC entries 2406074 and 2411703 contain the supplementary crystallographic data for CAU-52as (from 3D ED data) and CAU-52 (from PXRD data), respectively.

**Table 1:** Lattice Parameter and Crystallographic Data of CAU-52 Obtained from Rietveld Refinement^46^

	**CAU-52**
method	Rietveld
Crystal system	Cubic
Space group	*Pm**n* (#223)
*a* [Å]	21.2177(11)
α [°]	90
Volume [Å^3^]	9552(15)
Peak fit function	Pseudo-Voigt; Thompson-Cox-Hastings
Background function	Chebyshev polynomial
Number of refined parameters	44
*R*_wp_ [%]	4.12
*R*_Bragg_ [%]	2.51
GOF	2.89

### Crystal Structure Description

**Figure 2:**
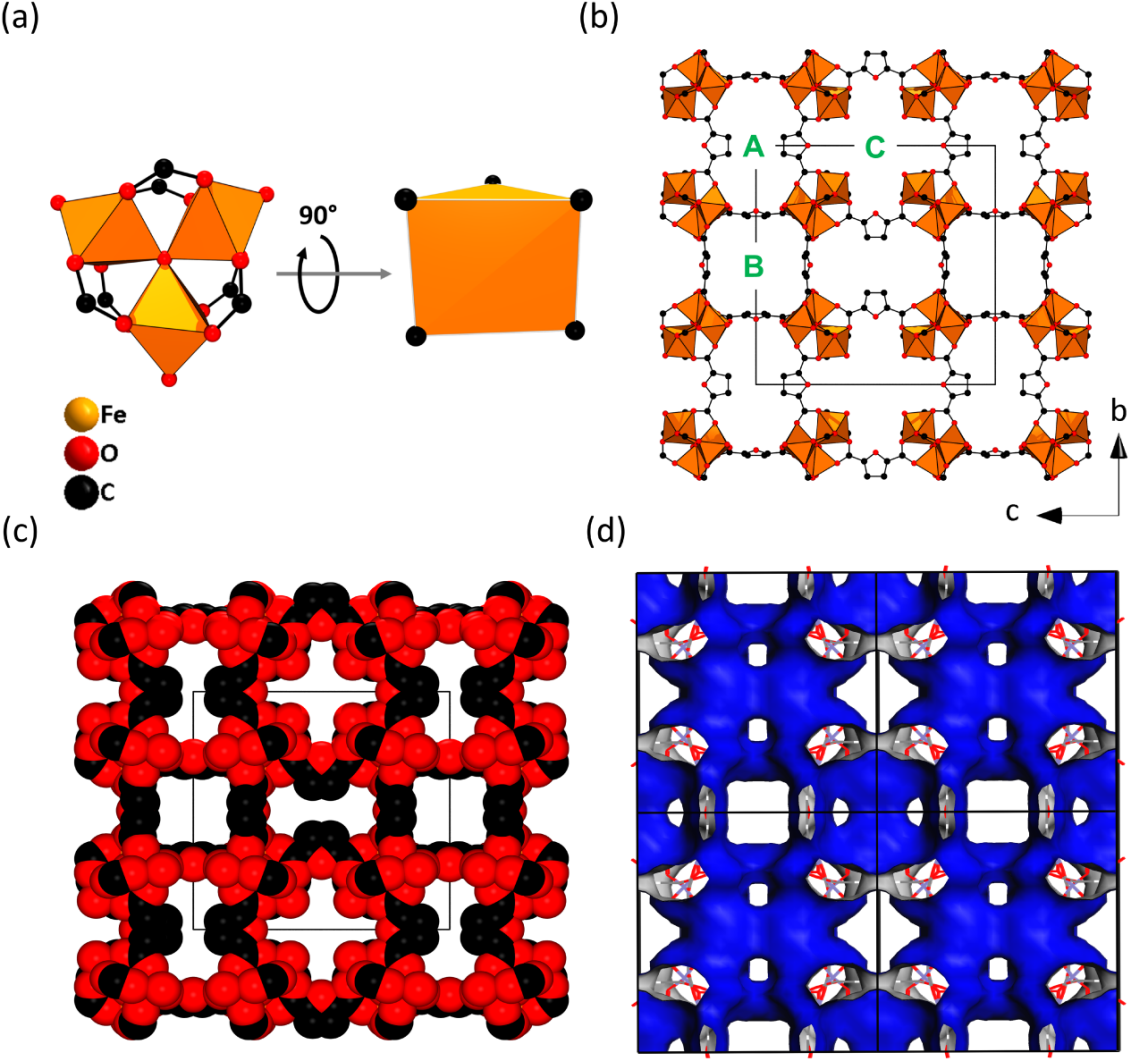
Crystal structure of CAU-52: (a) IBU and trigonal prismatic IBU, (b, c) 3D views of the pores along [100] as ball-and-stick and space-filling models, respectively. (d) Connolly surface along [100] modeled with Materials Studio^48^ (*r*(N_2_)=1.81 Å). The black lines indicate the unit cell edges.

Since the guest molecules in the pores of CAU-52 are disordered, they could not be localized from the electron or X-ray diffraction data. Hence, only the framework structure is described herein. CAU-52 crystallizes in the cubic space group *Pm**n* (#223) and contains trinuclear inorganic building units (IBUs) of corner-sharing FeO_6_ polyhedra joined by a μ_3_-O at their center (Figures 2a and S5). This IBU is well-known in Fe-MOF chemistry and is found, for example, in Fe-MIL-59,^10^ MIL-88,^34^ MIL-100,^3^ and MIL-101,^11^ Fe-soc-MOF (PCN-250^12^/MIL-127^35^), and PCN-333.^36^ The Fe^3+^ ions in the IBU are interconnected by six FDC^2–^ ion linker molecules, which represent the vertices of a trigonal prismatic IBU (Figure 2a). These IBUs are interconnected to six other IBUs, and a three-dimensional framework that carries the **pcu** net is formed. An In-MOF with the same ligand and the same framework topology was reported by Bu et al. in 2015.^24^

**Figure 3:**
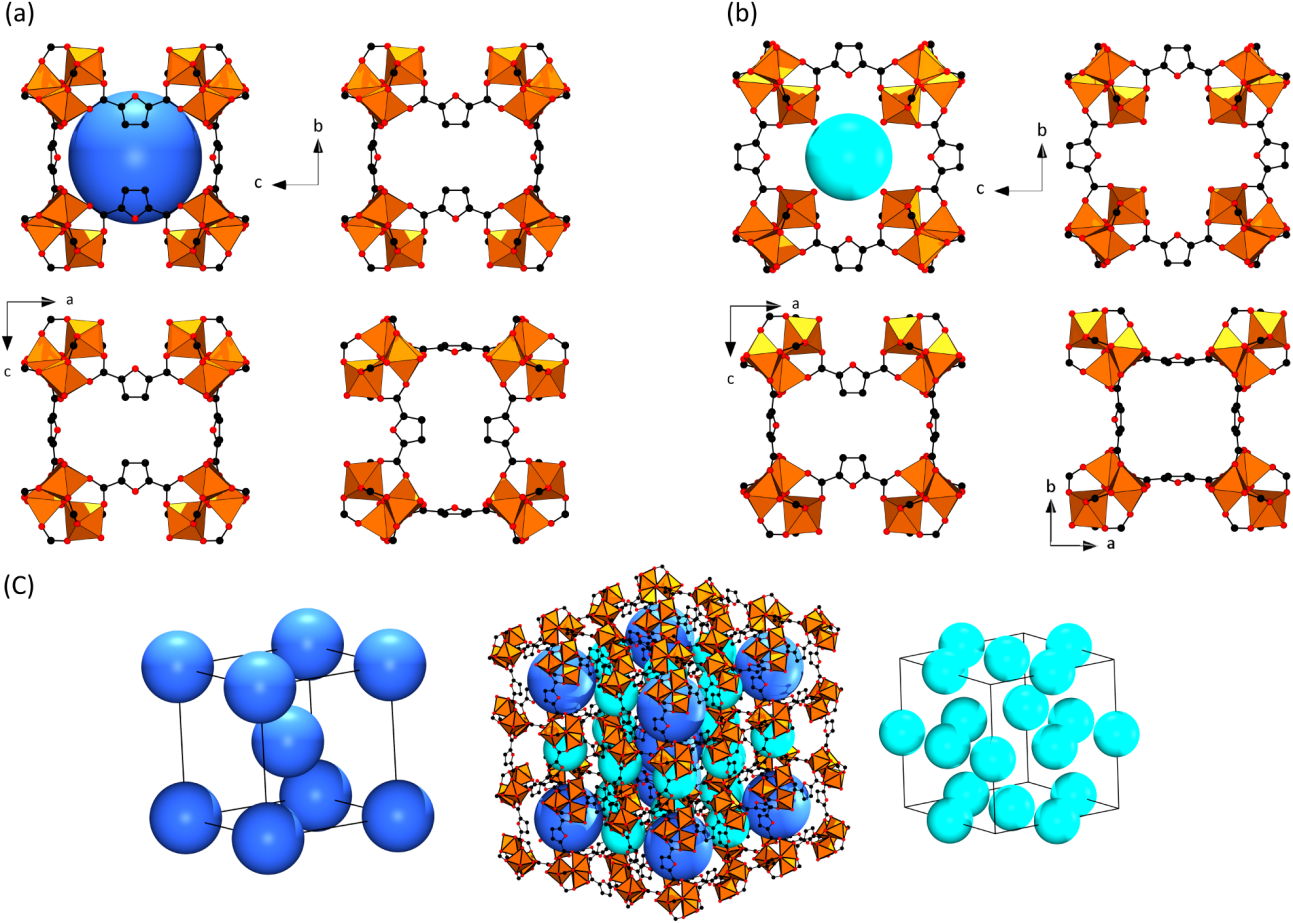
Pore system in the structure of CAU-52. (a, b) Two cubic building units can be used to describe the pore system. The dark blue sphere with a diameter of 5.0 Å corresponds to the cubic building unit that is a cage, while the cyan sphere with a diameter of 3.2 Å is located in the cubic building unit containing larger pore windows. (c) Arrangement of the cubic building units (dark blue and cyan) creates distinct channel types within the crystal structure, i.e., alternating dark blue and cyan spheres or exclusively cyan spheres. The diameter of the spheres was calculated by taking the van der Waals radii of the framework atoms into account.

Three distinct channels are observed, labeled with the bold green letters A, B, and C. The Connolly surface of the 3D pore system is visualized in Figure 2d. The structure of CAU-52 and the pore system are best described using two cubic building units Figure 3a,b. To distinguish the two, spheres in dark blue and cyan were added, corresponding to the available pore space. The first building unit, with the dark blue sphere, has trinuclear IBUs at the corners of the cube and FDC^2–^ ions on the edges (Figure 3a) and the furan rings of all the linkers in this building unit are parallel to the cube faces and point to the middle of the faces, leading to small pore windows; hence, we have called this cubic building unit a cage. This can best be seen by the projection of the cage along the *a*, *b*, and *c* axes (Figure 3a). The second building unit, with a cyan sphere shown in Figure 3b, also contains the trinuclear IBUs at the corners of the cube and the FDC^2–^ ions on the edges of the cube, but the orientation of the furan rings and the trimeric building units differs, leading to larger pore windows, which is reflected in the Connolly surface of the 3D pore system. Hence, this cubic unit is not denoted as a cage. The pore structure, with three distinct channel types, can now be understood by using these two cubic building units (Figure 3c). Alternation of the two cubic building units (dark blue and cyan) leads to channels denoted **A**, while channels **B** and **C** are constructed only by the second cubic building unit. The orientation of the cubes leading to channels **B** and **C** is marked in Figure 2 by the corresponding bold green letters.

The pore diameter of both cages was approximated to 3.2 and 5.0 Å by placing a sphere at the center of the cages and taking the van der Waals radii of the framework into account (Figure S6). The theoretical accessible surface area and the micropore volume of the pure framework without any guest molecules were calculated using Zeo++ with nitrogen (*r*(N_2_)=1.8 Å) as the probe molecule,^49,50^ resulting in values of *a*_s,BET_ = 1300 m^2^/g and *V*_mic_ = 0.25 cm^3^/g, respectively.

**Figure 4:**
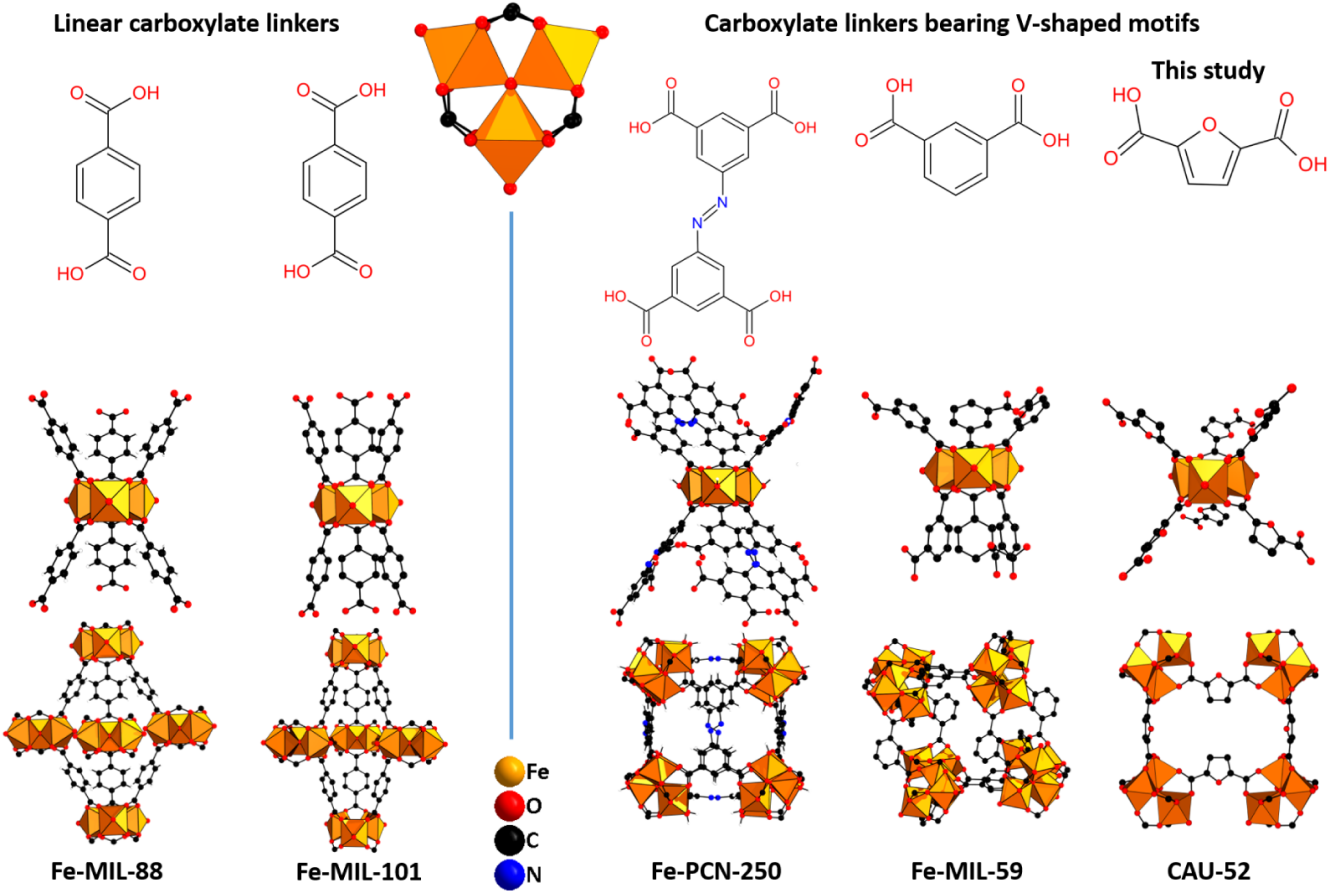
Comparison between the structures containing [Fe_3_(μ_3_-O)] clusters with linear linkers (left) and V-shaped linkers (right). The use of linear linker molecules leads to compounds denoted as Fe-MIL-88^34^ and Fe-MIL-101,^11^ crystallizing in a hexagonal space group. The use of V-shaped linkers or the tetracarboxylate linker 3,3′,5,5′-azobenzene tetracarboxylic acid, formally composed of two V-shaped linker molecules, leads to Fe-soc-MOF,^12,35^ Fe-MIL-59^10^ and CAU-52, which crystallize in cubic space groups.

The selection of linker molecules plays a central role in the structure and properties of Fe-MOFs, which are based on [Fe_3_(μ_3_-O)] clusters. As previously mentioned, most studies have focused on the use of linear dicarboxylic acids, while information on the use of V-shaped dicarboxylate linkers is relatively scarce. The symmetry of the organic linker molecules and their coordination modes are crucial for the formation of highly ordered structures.^51,52^ For example, the connection of the tetracarboxylate linkers in Fe-soc-MOF (H_4_ABTC = 3,3′,5,5′-azobenzenetetracarboxylic acid),^12^ the V-shaped linkers in MIL-59 (isophthalic acid = m-H_2_BDC),^10^ and CAU-52 (2,5-furandicarboxylic acid = H_2_FDC) with trinuclear [Fe_3_(μ_3_-O)] clusters favors the formation of highly symmetric structures crystallizing in cubic space groups (Figure 4).

**Figure 5:**
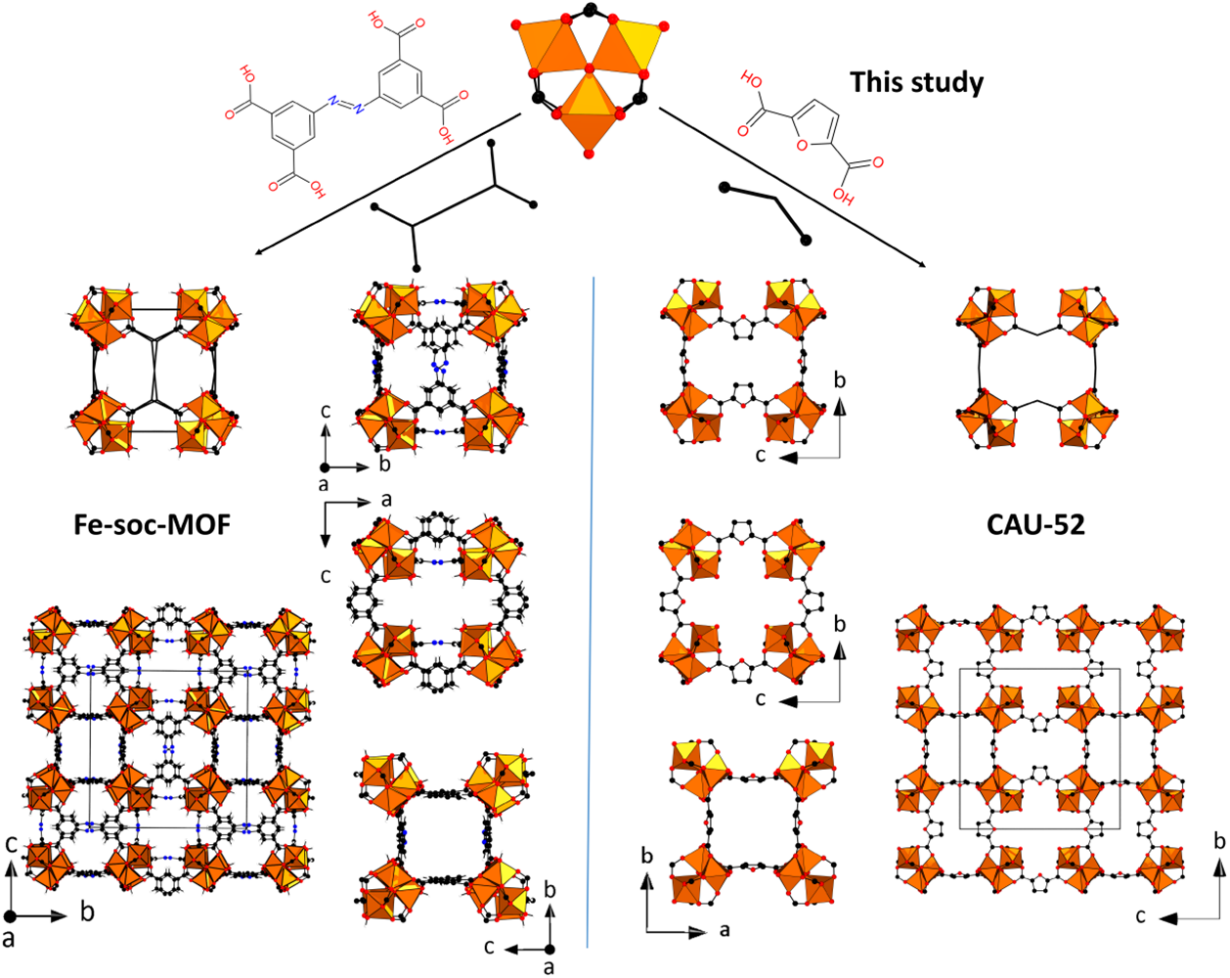
Comparison of the crystal structure of Fe-soc-MOF (PCN-250^12^/MIL-127^27^) (left) and CAU-52 (right).

Figure 5 shows a comparison between the structures of CAU-52 and Fe-soc-MOF (PCN-250^12^/MIL-127^35^). In Fe-soc-MOF, each rectangular tetratopic carboxylate linker connects four [Fe_3_(μ_3_-O)] clusters, while in CAU-52, each linker bridges only two [Fe_3_(μ_3_-O)] clusters. In both structures, very similar arrangements of two types of cubic cages are found.

### Characterization

**Figure 6:**
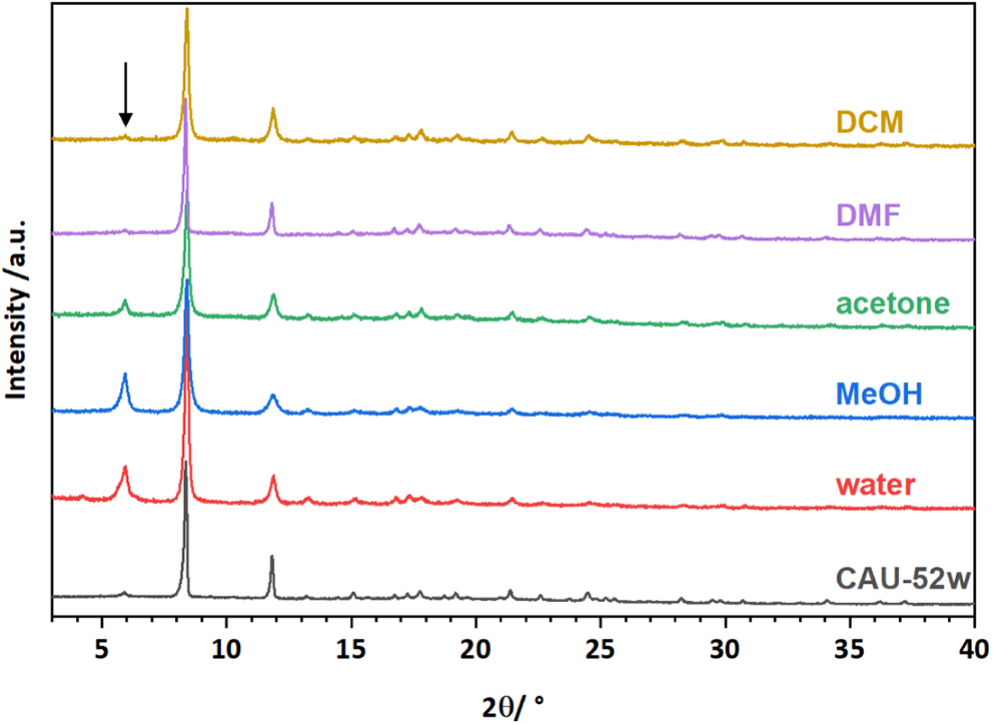
PXRD patterns of CAU-52 after 24 h of stirring in dichloromethane (DCM), dimethylformamide (DMF), acetone, methanol (MeOH) and water (pH = 6) at room temperature.

The chemical stability of CAU-52 in various organic solvents and water was investigated by stirring the material in different solvents for 24 h (approximately 5 mg/mL). Subsequently, the samples were centrifuged, dried at 70 °C for 1.5 h in a drying oven, and characterized by PXRD (Figure 6). The results demonstrate high stability in organic solvents and in water at pH 6, but a change in the relative intensities. This is most prominently visible for the 110 Bragg reflection at approximately 2θ = 6° (marked with a black arrow) and can be explained by the different electron densities and adsorption sites of the guest molecules. In addition, peak broadening is also detected, which is due to the loss of long-range order.

The composition of CAU-52 was determined using a series of characterization methods. The elemental analysis of CAU-52 confirms the absence of residual DMF molecules that was used to remove recrystallized linker. Accordingly, elemental analysis gives values matching well to a composition [Fe_3_(μ_3_-O)(FDC)_3_(OH)(H_2_O)_2_]·5H_2_O·H_2_FDC, including a free linker molecule (C (obs. 30.28%, calc. 30.50%), H (obs. 3.05%, calc. 2.67%), and N (obs. 0)).

In order to analyze the dehydration process and the host–guest interactions of CAU-52, variable-temperature diffuse reflectance Fourier transform infrared spectroscopy (VT-DRIFTS) was employed. The sample was gradually heated in air from room temperature to 360 °C. Prior to conducting the VT-DRIFTS measurements, the sample was stored in a sealed desiccator at 85% relative humidity for 3 d in order to ensure full hydration. This sample was designated as CAU-52_85%_R.H. (85% relative humidity) for further analysis. The PXRD pattern after exposure to 85%_R.H. is presented in Figure S7. Further details can be found in Section S3.1.

**Figure 7:**
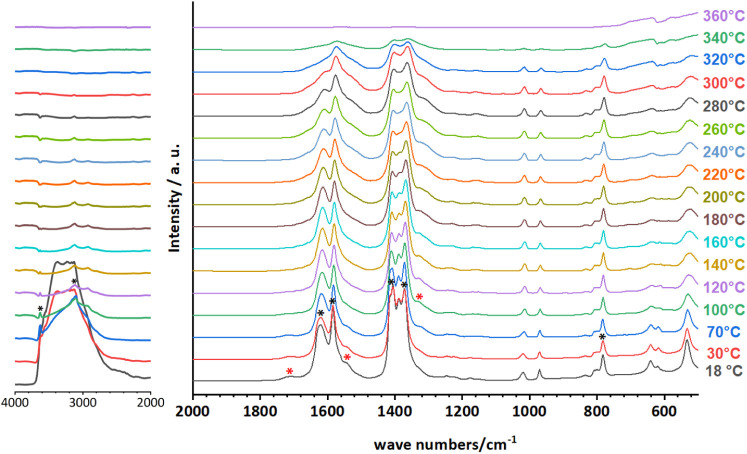
VT-DRIFTS spectra of CAU-52 collected between 18 and 360 °C. The sample was stored in a desiccator under 85% relative humidity for 3 d prior to the measurement. Bands of the linker as part of the framework are marked with black asterisks. Bands of the H_2_FDC/FDC^2–^ molecules in the pores are marked with red asterisks. The complete spectra from 500 to 4000 cm^–1^ are presented in Figure S9.

The VT-DRIFTS (Figure 7) and ATR-MIR (Figure S8) spectra both display intense broad bands observed between 3700 and 2500 cm^–1^ originating from H_2_O molecules in the pores of CAU-52. The corresponding deformation vibration of H_2_O at 1545 cm^–1^ (marked with a red asterisk) is clearly visible only in the DRIFTS spectrum,^53^ in which a band at 3630 cm^–1^ is visible from coordinating OH^–^ ions. The asymmetric and symmetric C–O stretching vibrations of the coordinating carboxylate groups of the framework can be assigned to the bands with maxima at 1620 cm^–1^ and 1407 cm^–1^, respectively.^27^ Additionally, bands corresponding to the ring vibrations of the furan unit are observed at 1584 cm^–1^ and 1371 cm^–1^.^27^ The −C–H deformation vibration of the aromatic furan ring occurs at 784 cm^–1^.^54^ These vibration bands are marked with asterisks and are listed in Table S5. Comparison with the spectrum of the protonated linker (H_2_FDC) led to the assignment of an additional band at 1715 cm^–1^, originating from the C=O stretching vibration of the furandicarboxylic acid groups (Figure 7, red asterisk).^55^

It can be observed that the heating process up to 120 °C results in a decrease of the intensity or the complete disappearance of the bands of the physisorbed and coordinating water molecules (broad, 3700–2500 cm^–1^) and the free protonated linker (1715 cm^–1^, Figure 7, red asterisk). Concomitantly, the band at 1325 cm^–1^ (red asterisk), which can be attributed to the symmetric vibration of the carboxylate group (−COO^–^), increases in intensity.^56^ These observations suggest that the carboxylic acid groups of the free linker molecules condense during heating and form acid anhydride groups.^57^

The presence of H_2_FDC in the pores of CAU-52 was also confirmed by ^1^H NMR spectroscopy using sodium formate (NaO_2_CH) as an internal standard (see Section S3.3). First, a calibration curve was recorded using three samples containing defined amounts of H_2_FDC and NaO_2_CH by determining the relative intensities of the ^1^H NMR peaks at 6.62 and 8.08 ppm (Figure S10 and Table S6). Subsequently, the NaO_2_CH standard and 5.5 mg of CAU-52 were dissolved in 10% NaOD/D_2_O and the reddish-brown iron hydroxide precipitate was separated by centrifugation. The ratio of the observed signals of the linker to NaO_2_CH (3.79) corresponds to 3.24 mg of linker, which corresponds to 59% of the total mass of CAU-52 and the composition [Fe_3_(μ_3_-O)(FDC)_3_(OH)(H_2_O)_2_)]·5H_2_O·H_2_FDC. Thus, the results of the elemental analysis and the TG measurements, which are presented in the following section, are confirmed.

### Thermal Properties

**Figure 8:**
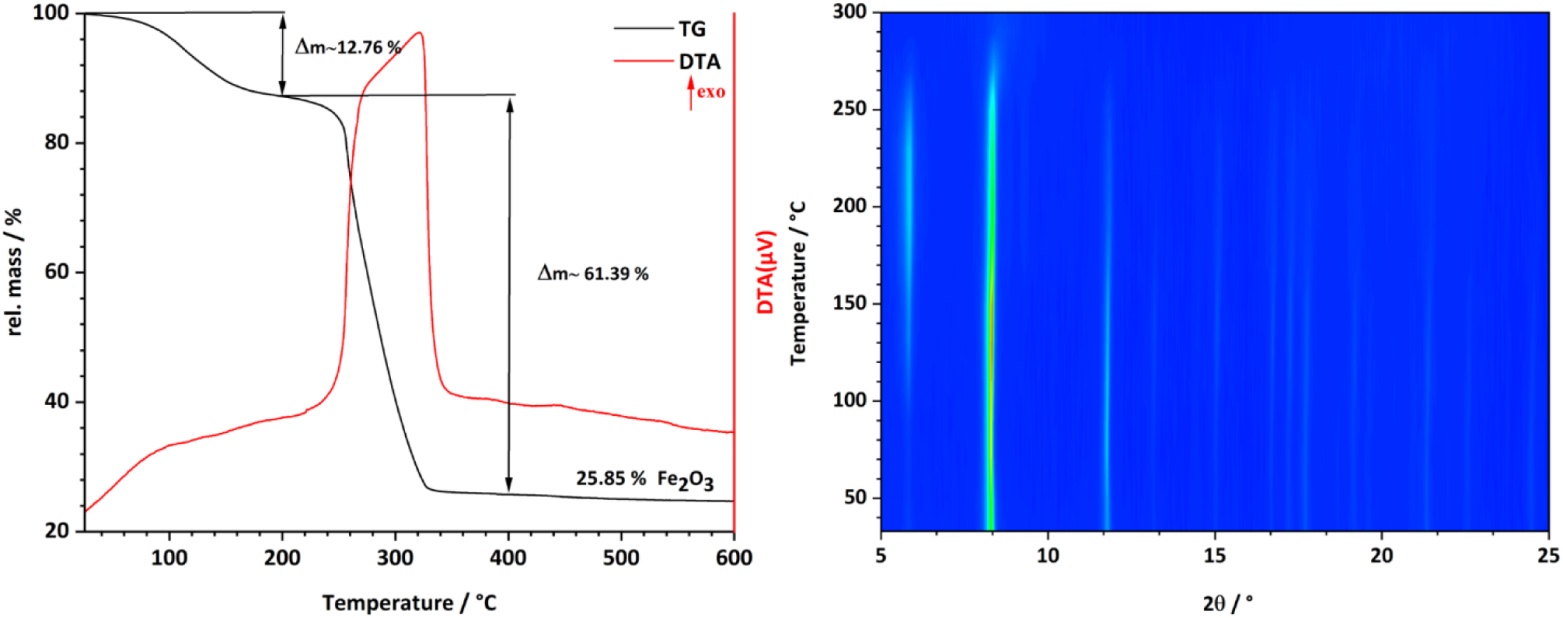
TG curve (left) and contour plot of VT-PXRD measurement (right) of [Fe_3_(μ_3_-O)(FDC)_3_(OH)(H_2_O)_2_]·5H_2_O·H_2_FDC (CAU-52).

Thermal properties of CAU-52 were studied by thermogravimetric analysis and variable-temperature (VT) PXRD. The TG curve is shown in Figure 8 (left), and two mass losses are observed. The initial mass loss, occurring between 50 and 200 °C, is approximately 12.76% and is attributed to the release of seven water molecules per formula unit (calc.:13.35%). The second strongly exothermic mass loss, between 200 and 400 °C (61.39%, calcd 61.31%), can be attributed to the degradation of the framework and the H_2_FDC molecules, as well as the formation of Fe_2_O_3_, which was confirmed by PXRD (Figure S11). The results of the TG analysis are in good agreement with the formula [Fe_3_(μ_3_-O)(FDC)_3_(OH)(H_2_O)_2_]·5H_2_O·H_2_FDC. The VT-PXRD patterns of CAU-52 are shown in Figure 8 (right). Only minor shifts in the reflection positions are observed, showing the rigidity of the framework. Nevertheless, strong changes in the relative intensities are found, which can be explained by the release of guest molecules from the pores, and at elevated temperatures (around 250 °C), framework decomposition occurs.

### Sorption Properties

**Figure 9:**
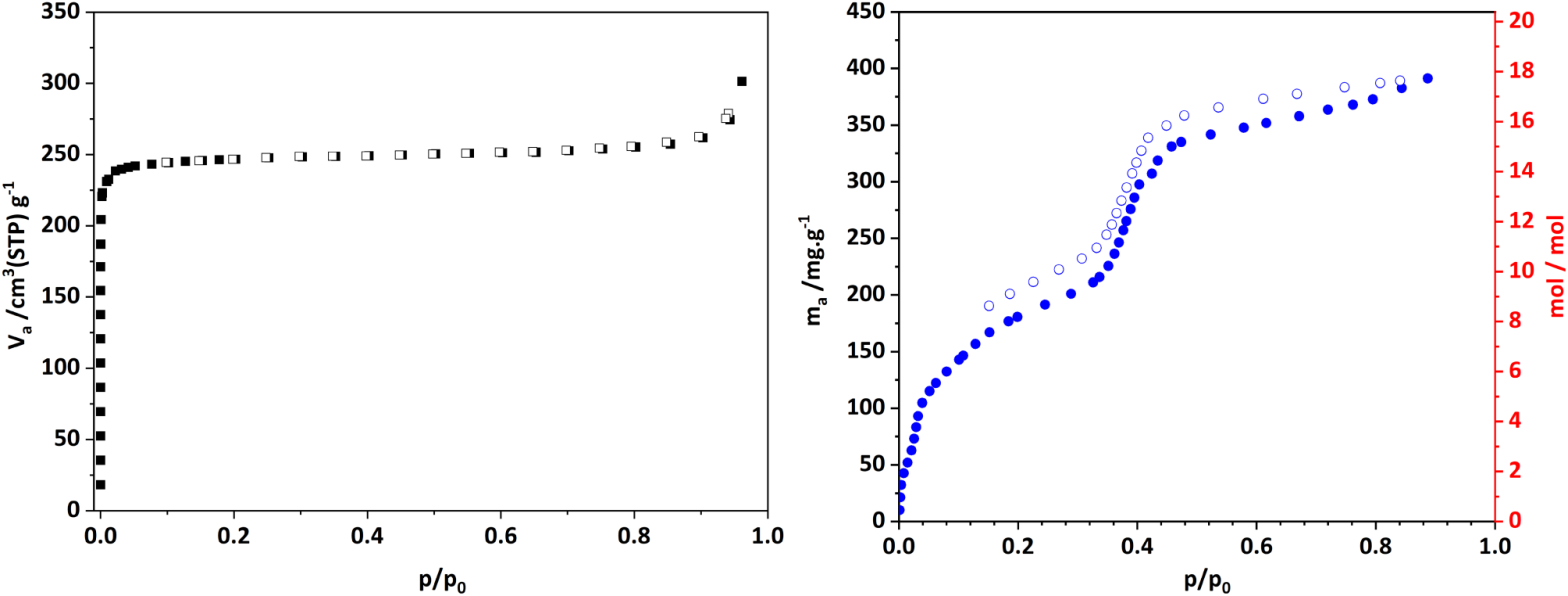
Nitrogen sorption isotherms (squares, left) and water vapor sorption isotherms (circles, right) of CAU-52 measured at 77 and 298 K, respectively. Filled symbols represent adsorption, and empty symbols desorption.

The sorption properties of CAU-52 were investigated by using nitrogen and water as adsorbates at 77 K and 298 K, respectively, to determine the specific surface area and water uptake (Figure 9). Prior to the measurements, the samples were treated for 16 h at 100 °C under reduced pressure (10^–2^ kPa) to remove adsorbed water molecules. PXRD measurements of the samples after the sorption experiments confirmed the crystallinity and stability of the samples, although a decrease in long-range order was observed (Figure S13).

The N_2_ sorption isotherm of CAU-52 exhibits a type I shape according to the International Union of Pure and Applied Chemistry (IUPAC), which is indicative of microporous substances.^58^ The specific surface area of *a*_s,BET_ = 1074 m^2^/g was determined using the Rouquerol method (Figure S14).^59^ This value is below the theoretical accessible surface area (ASA) of 1300 m^2^/g calculated with nitrogen (*r*(N_2_)=1.8 Å) using Zeo++.^49,50^ This reduced specific surface area can be attributed to the presence of adsorbed linker molecules, which were not taken into account in the theoretical calculations.

The water sorption isotherm exhibits three steps, with the first one corresponding to two water molecules per formula unit (below *p*/*p*_0_ < 0.04). This step can be assigned to the two coordinating water molecules. The second step, up to *p*/*p*_0_ = 0.2, corresponds to a total uptake of eight water molecules per formula unit, and the third large step, corresponding to an uptake of almost ten more water molecules at *p*/*p*_0_ = 0.89, is observed for CAU-52. This corresponds to a water uptake of 390 mg/g, corresponding to 18 mol of H_2_O per formula unit but it decreases slightly in subsequent sorption cycles (Figure S15). The shape of the water isotherm is influenced by the presence of free metal coordination sites, the pore size, and the ability to form hydrogen-bonded networks of water molecules.^10,30,31,60^ To further study the high water uptake of molecules per formula unit, a sample of CAU-52 stored for 3 days at 85% relative humidity (see VT-DRIFT spectroscopy) was also analyzed by TG (Figure S12 and Table S7) and elemental analysis (Table S4). Both characterization methods support the results of the water sorption measurements, and due to the extended treatment time in humidified air, an uptake of 20 molecules per formula unit is observed. Although CAU-52 has a high water uptake in the range of 0–50% relative humidity, the shape of the isotherm is not ideal to make it a suitable candidate for water harvesting applications.

## Conclusion

A new Fe-MOF with the composition [Fe_3_(μ_3_-O)(FDC)_3_(OH)(H_2_O)_2_]·5H_2_O·H_2_FDC (CAU-52) was obtained using furan dicarboxylic acid as a renewable building block in water under solvothermal conditions. The crystal structure was determined through 3D electron diffraction and further refined using Rietveld analysis against PXRD data. The structure of CAU-52 contains the well-known trinuclear [Fe_3_(μ_3_-O)] clusters, which are connected by furandicarboxylate ions to form a three-dimensional framework exhibiting structural similarities with Fe-soc-MOF (PCN-250^12^/MIL-127^35^). Various characterization methods were used to confirm the composition. Thus, CHN analysis, quantitative ^1^H NMR spectroscopy, and thermogravimetric analysis confirmed the presence of adsorbed linker molecules in the pores, which cannot be removed even by extensive washing with organic solvents. CAU-52 is porous toward N_2_ and H_2_O, and variable temperature PXRD revealed thermal stability up to 250 °C, which is consistent with the results of the TG measurements. High water uptake was observed in water adsorption at 298 K at high relative humidity values, which was also confirmed by TGA and elemental analysis.

## Supporting Information Available

Additional experimental details, PXRD patterns of compounds obtained in screening experiments and after the washing procedure, details on the Rietveld refinement, a detailed description of the crystal structure, ^1^H NMR, IR and DRIFT spectra, results of CHN analyses, the TG curve of CAU-52_85%_R.H. and PXRD data of the residue after TG measurements (PDF)
Adsorption information files (TXT)
Crystallographic data (TXT)

(PDF)
(TXT)
(TXT)
