# Supplementary material for: CAU-52: An Iron Metal–Organic Framework Containing Furandicarboxylate Linker Molecules
Source: Inorg Chem. 2025 Apr 7;64(15):7450–9. doi: 10.1021/acs.inorgchem.5c00184 (PMC12015821; doi:10.1021/acs.inorgchem.5c00184)
Supplement: Supplementary file 1 — ic5c00184_si_001.pdf [file ic5c00184_si_001.pdf]

Supporting information

CAU-52: An Iron Metal-Organic Framework Containing Furandicarboxylate Linker Molecules

Essam Alkhnaifes[a], Erik Svensson Grape[b,c,], A. Ken Inge[b], Felix Steinke[a], Tobias A. Engesser[a] and Norbert Stock[a,*]*

[a]Institut für Anorganische Chemie, Christian-Albrechts-Universität zu Kiel, 24098 Kiel, Germany. E-Mail: stock@ac.uni-kiel.de

[b]Department of Materials and Environmental Chemistry, Stockholm University, Stockholm 10691, Sweden.

[c]E. Svensson Grape: Current address: Department of Chemistry-Angström Laboratory; Synthetic Molecular Chemistry, Uppsala University, 75120 Uppsala, Sweden.

E-Mail: erik.svensson-grape@kemi.uu.se

Table of content

1. Synthesis ... 3
   1.1. Investigation of the system $Fe^{3+}/H_2FDC/NaOH/water/co-solvent$ ... 3
   1.2. Synthesis of CAU-52as and formation of CAU-52 ... 4
2. Crystal structure ... 5
   2.1. Structure determination of CAU-52as and CAU-52 ... 5
   2.2. Structure description of CAU-52 ... 6
3. Characterization ... 8
   3.1. CHN analysis ... 8
   3.2. ATR-MIR, VT-DRIFTS spectroscopy and PXRD of CAU-52 ... 8
   3.3. Quantitative determination of the linker content in CAU-52 ... 11
   3.4. TG analysis ... 13
   3.5. Sorption studies ... 14
4. References ... 16

1. Synthesis

1.1. Investigation of the system $Fe^{3+}/H_2FDC/NaOH/water/co\text{-}solvent$

Initially the use of three different sources of Fe(III) was investigated and the molar ratio Fe^{3+} : H_2FDC : NaOH was kept constant at 1 : 1 : 9. In addition to acetic acid as the co-solvent, the influence of DMF was investigated. The syntheses were carried out in 2 ml Teflon inserts in a 24-multiclave with the temperature time program 120 °C / 1 h - 6 h - 3 h. The amount of the above reactants are listed in Table. S1 and the recorded PXRD patterns of the reaction products are shown in Fig. S1. Solution of the following concentrations were used: c(Fe^{3+}) = 0.5 mol/L, c(Na_2FDC) = 0.5 mol/L.

Table S1: Details on the screening investigation of the system $Fe^{3+}/H_2FDC/NaOH$/water/co-solvent using different Fe(III) salts as well as AcOH and DMF as co-solvents. A = amorphous reaction product.

Metal Salt	#.	mmol Linker : Fe^{3+}		Na_2FDC [µL]	AcOH [µL]	DMF [µL]	H_2O [µL]	Fe^{3+} aq [µL]	result
$FeCl_3 \cdot 6H_2O$	1	0.4	0.36	800	480	-	-	720	CAU-52
$Fe(NO_3)_3 \cdot 9H_2O$	2	0.4	0.36	800	480	-	-	720	CAU-52
$Fe_2(SO_4)_3$	3	0.4	0.36	800	480	-	360	360	CAU-52
$FeCl_3 \cdot 6H_2O$	4	0.4	0.36	800	-	480	-	720	A
$Fe(NO_3)_3 \cdot 9H_2O$	5	0.4	0.36	800	-	480	-	720	A
$Fe_2(SO_4)_3$	6	0.4	0.36	800	-	480	360	360	A

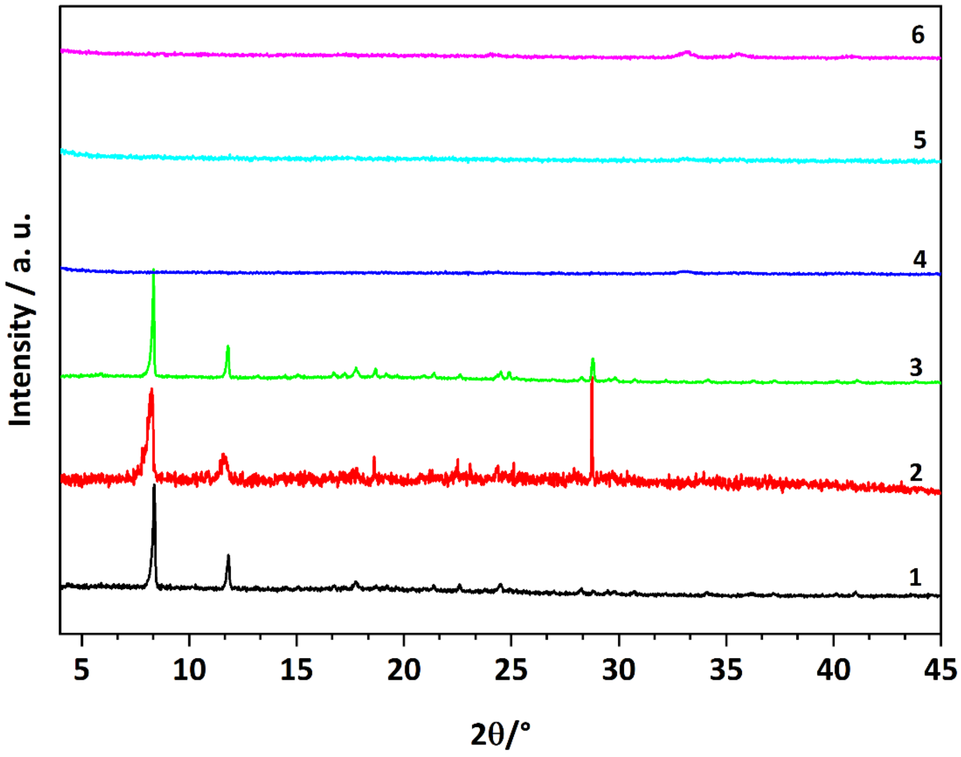

Fig. S1: PXRD patterns of the reaction products obtained in the screening (Table S1).

Reactions in DMF as the co-solvent lead to the formation of X-ray amorphous products, while the use of AcOH results in the formation of crystalline materials (Fig. S1).

1.2. Synthesis of CAU-52as and formation of CAU-52

To obtain a large quantity of CAU-52 for further detailed characterization, the reaction was carried out six times. The PXRD patterns of the reaction products are shown in Fig. S2.

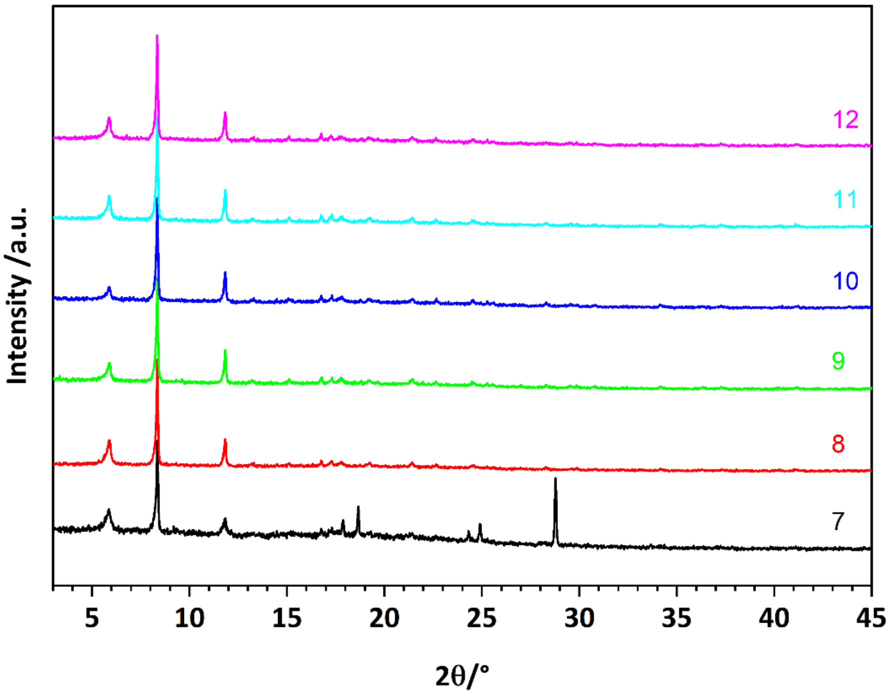

Fig. S2: PXRD patterns of the reaction products obtained using identical reaction conditions six times, in order to get larger amounts of **CAU-52as** for further detailed characterization. The additional reflections observed in the PXRD pattern of sample 7 are due to the presence of crystalline linker. This crystalline linker was removed by in a subsequent washing step.

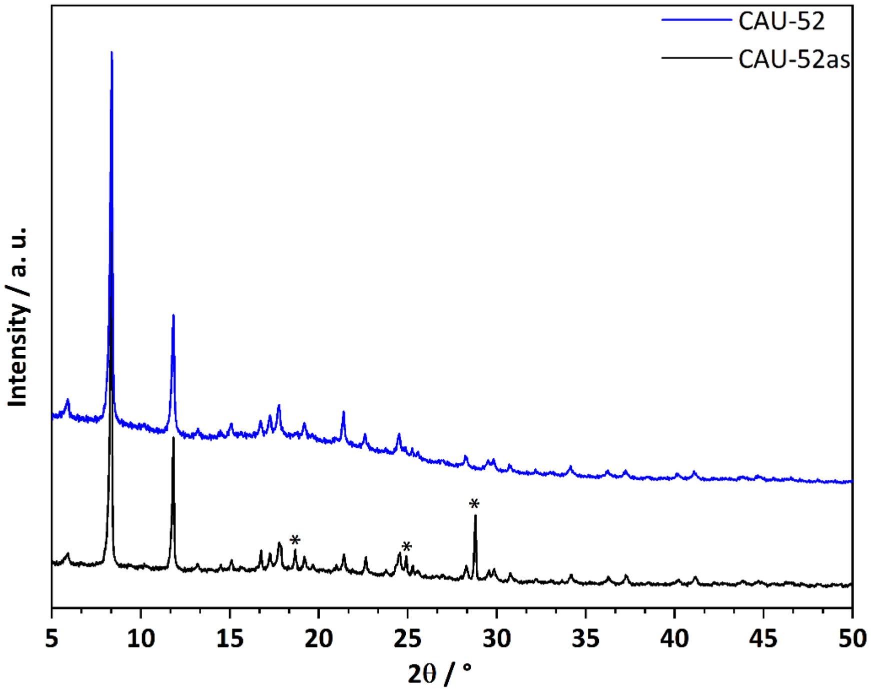

Fig. S3: PXRD patterns of CAU-52as(black) and $[Fe_3(\mu_3\text{-}O)(FDC)_3(OH)(H_2O)_2]\cdot 5\ H_2O\cdot H_2FDC$, CAU-52 (blue) obtained after washing the sample with DMF and H_2O. The reflections of the linker observed in the as synthesized sample are marked with an asterisk.

2. Crystal structure

2.1. Structure determination of CAU-52as and CAU-52

The crystal structure of CAU-52as was determined through three-dimensional electron diffraction (3D ED) measurements.

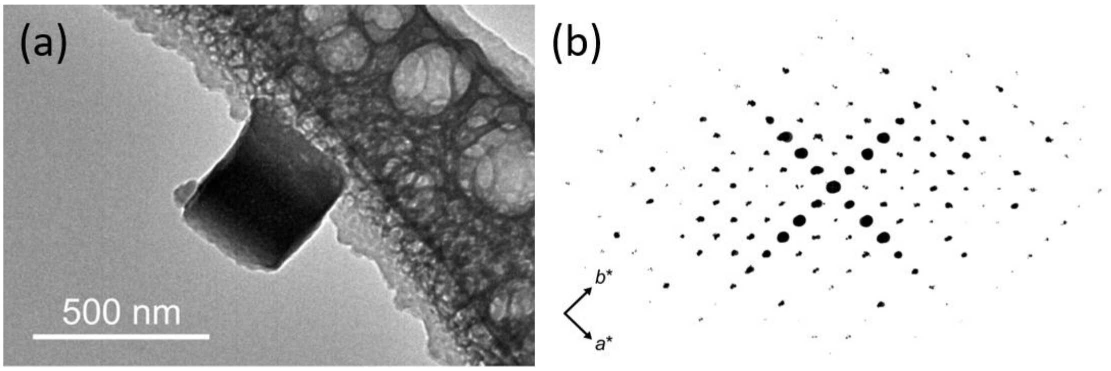

Fig. S4. (a) TEM-micrograph of CAU-52as and (b) reconstructed reciprocal space projection viewed along *c**. from three-dimensional electron diffraction data (3D ED).

Table S2. Lattice parameter and crystallographic data of CAU-52as obtained from 3D ED data.

	CAU-52as
method	3D ED
crystal system	cubic
space group	$Pm\bar{3}n$ (#223)
a [Å]	21.974(3)
α [°]	90
volume [$Å^3$]	10610(4)
R_1 [%]	23.3
R_2 [%]	53.1
GOF	0.927

The crystal structure obtained from 3D electron diffraction data was used as starting model for a Rietveld refinement as implemented in Topas Academic[1]. For the refinement, a PXRD pattern was collected and indexed to obtain the lattice parameters for the model. The atoms Fe1, O3, O4 and O5 were fixed to special positions, while the remaining atoms were refined on general positions. Guest molecules were considered and refined as oxygen atoms. The guest molecules O01, O02 and O04 were fixed to special positions inside the pores to account for smeared electron density, but avoid merging of partially occupied oxygen atoms when doing the refinement with the respective atoms on general positons.
The bond lengths and angles of the linker molecule and the inorganic building unit were refined using distance and angle restraints and are in good agreement with data of other Fe(III) carboxylates listed in the MOF subset[2] of the CSD, which were analysed using the software Conquest and Mercury[3].
The final parameters obtained from the Rietveld refinement are summarized in Table 1 and the Rietveld plot is shown in Fig. 1 (left) in the manuscript.

2.2. Structure description of CAU-52

The crystal structure of CAU-52 was refined by the Rietveld method. The asymmetric unit of CAU-52, as illustrated in Fig. S5a, consists of half an FDC^{2-} (a FDC^{2-} ion on a mirror plane) and one Fe^{3+} ion. The Fe^{3+} ion is coordinated by three oxygen atoms, one μ_3-O^{2-} ion (O4), one of FDC^{2-} ion (O1) and one coordinated water molecule (O3). The inorganic building unit (IBU) is formed by three Fe^{3+} ions (Fe1) that are bridged via a μ_3-O^{2-} ion (O4) and each Fe^{3+} ion is coordinated by carboxylate groups of four different FDC^{2-} ions (Fig. S5b). The coordination spheres are completed by two coordinated water molecule and hydroxyl ion for charge balance. Each IBU is connected to six other IBUs through the FDC^{2-} ions (Fig. S5c). The interconnection of IBUs through linker molecules results in the formation of a cubic shaped three dimensional framework (Fig. 2 in the manuscript). This interconnection results in the formation of three-dimensional pore system that is filled with guest molecules. Selected bond lengths for the structure model of CAU-52 are listed in Table S3.

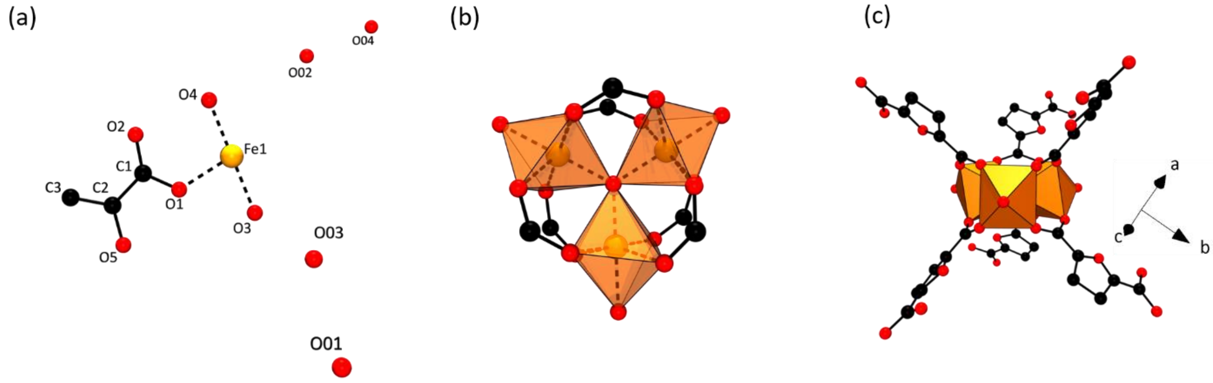

Fig. S5. Crystal structure of CAU-52 (a) asymmetric unit (b) IBU and (c) IBU with six FDC^{2} ions.

Table S3. Bond lengths observed in CAU-52 as obtained from Rietveld refinement.

Atoms #1	Atoms #2	Distance / Å
**Fe1**	O1	2.00(5)
	O2	1.96(4)
	O3	1.97(5)
	O4	1.901(13)
**C1**	O1	1.29(9)
	O2	1.28(8)
	C2	1.44(9)
**C2**	O5	1.33(7)
	C3	1.37(7)

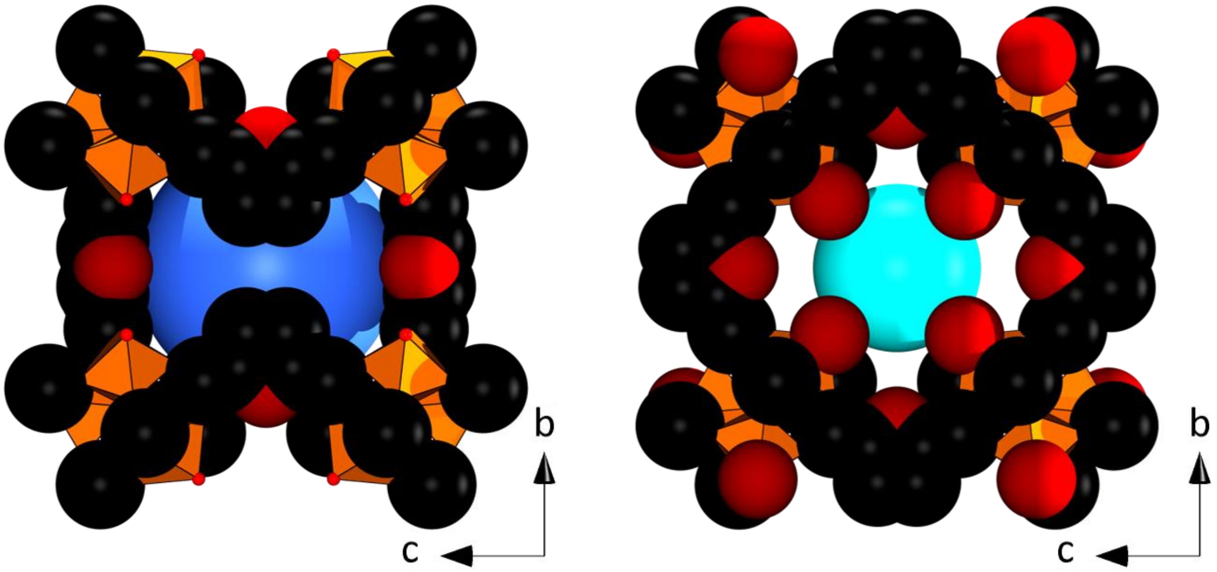

Fig. S6 Pore diameter of the two cubic building units. The dark blue sphere with a diameter of 5 Å represents the cubic cage-like building unit (left) and the cyan sphere with a diameter of 3.2 Å is positioned within the cubic building unit containing larger pore windows (right).

3. Characterization

To confirm the composition of CAU-52 and a sample equilibrated at 85 % relative humidity (denoted CAU-52_85%_R.H.), various characterization methods were employed, including CHNS analysis, ATR-IR and VT-DRIFT spectroscopy, ^{1}H NMR spectroscopy, thermogravimetric (TG) analysis and N_2 and H_2O sorption.

3.1. CHN analysis

The results of the CHN analysis of a CAU-52 sample equilibrated at 85 % relative humidity (denoted CAU-52_85%_R.H.) is presented in Table S4. The results of CAU-52 stored under ambient conditions are presented in the manuscript.

Table S4. The results of CHN analysis of $[Fe_3(\mu_3\text{-}O)(FDC)_3(OH)(H_2O)_2]\cdot 18H_2O\cdot H_2FDC$ (CAU-52_85%_R.H.).

sample	**C [%]**		**H [%]**		**N [%]**
	obs.	calc.	obs.	calc.	obs.
CAU-52_85%_R.H.	23.89	24.45	4.36	4.36	0

3.2. ATR-MIR, VT-DRIFTS spectroscopy and PXRD of CAU-52

A MIR-spectrum of **CAU-52** was collected on a Bruker ALPHA-FT-IR A220/D-01 using an ATR-unit and is shown in Fig. S8. The most important vibrational bands are listed in Table S5.
In addition, the PXRD patterns of **CAU-52**, both before and after exposure to 85% relative humidity, are shown in Fig. S7. The resulting VT-DRIFT spectra are shown in Fig. S9. Prior to conducting the VT-DRIFTS measurements, the sample was stored for three days in a sealed desiccator at a relative humidity of 85%[4] to ensure uniform and stable moisture distribution. The sample denoted as CAU-52_85%_R.H. (85% relative humidity) was placed on a mesh platform in the upper section of the desiccator, while a saturated KCl solution was placed in a dish in the lower section to avoid direct contact between the sample and the solution. CAU-52_85%_R.H. was taken directly from the desiccator and diluted with sufficiently dried KBr. Subsequently, the sample was heated in air up to 370 °C in 20 °C increments, with complete decomposition observed above 350 °C.

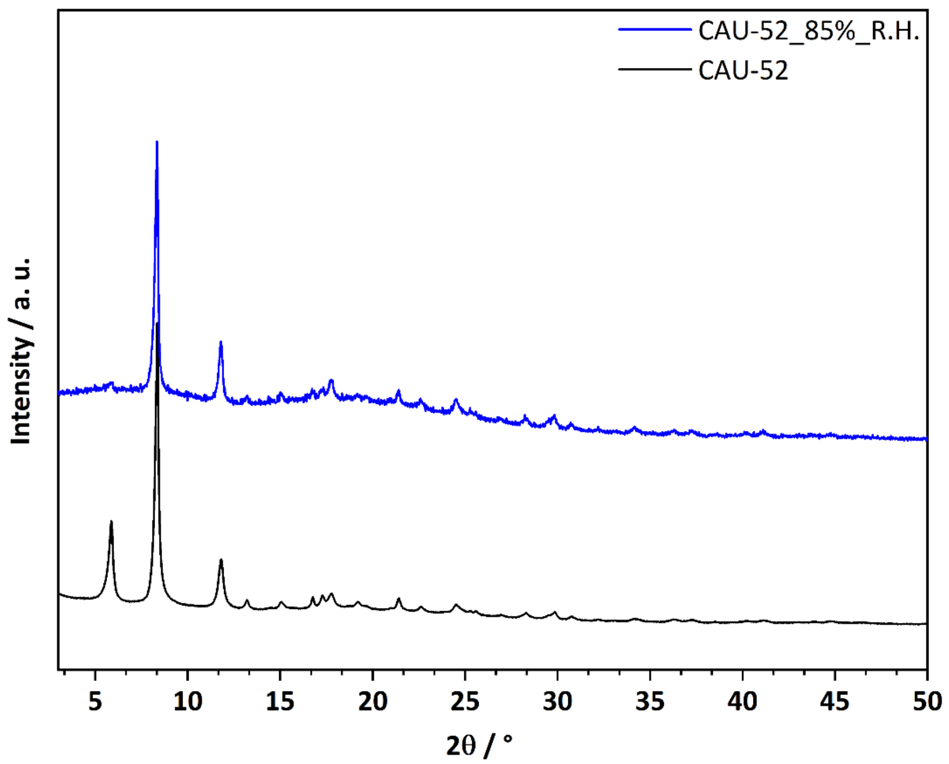

Fig. S7: PXRD patterns of CAU-52 before and after exposure to 85% relative humidity for 3 days.

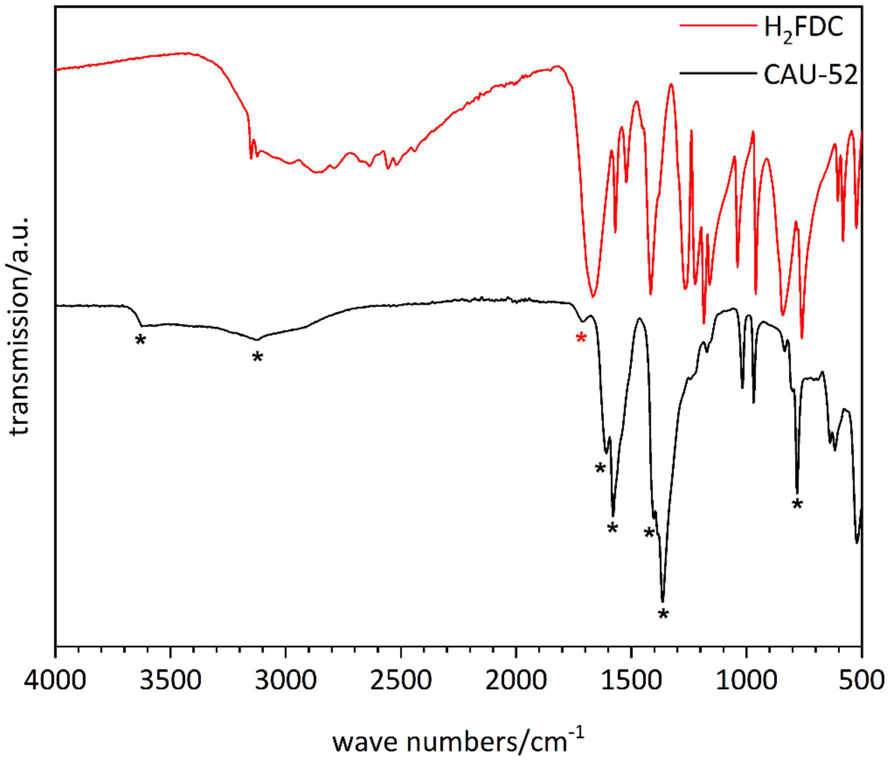

Fig. S8. MIR spectrum of CAU-52. Bands at 3630, 3125, 1715, 1620, 1584, 1407, 1371, 785 cm^{-1} marked with an asterisk are discussed in the manuscript. IR spectrum from the bulk linker molecules is added for comparison.

Table S5. Assignment of the vibrational bands in IR-spectrum of CAU-52.

**Infrared Band /cm^{-1}**	**Vibration**
3630	ν_s(OH)
3125	ν_s(CH) (aromat.)
1715	ν(C=O) (free –COOH)
1620	ν_{as}($-CO_2^-$)
1584	ring vibration (furan)
1407	ν_s($-CO_2^-$)
1371	ring vibration (furan)
785	δ(C-H)

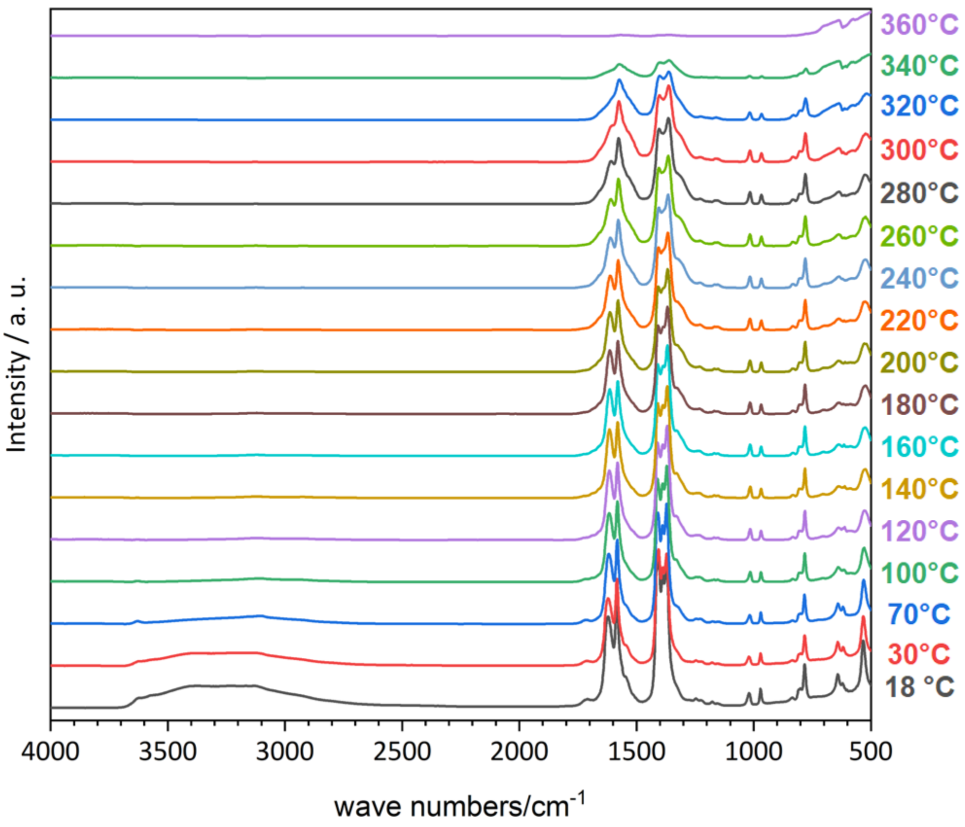

Fig. S9. Complete VT-DRIFT spectra of CAU-52 collected at temperatures between 18 and 360 °C. The sample was stored at 85 % relative humidity for 3 days prior to the measurements.

3.3. Quantitative determination of the linker content in CAU-52

For the quantitative determination of the linker content in CAU-52, a stock linker solution was prepared by dissolving 2,5-furandicarboxylic acid (31.2 mg, 0.2 mmol) in 10 % NaOD/D_2O (1 mL volumetric flask). Sodium formate (NaO_2CH) (7 mg, 0.1 mmol) was dissolved in 2 mL of 10 % NaOD/D_2O as an internal standard reference. Three defined concentrations, denoted as C_1 (0.01 mmol), C_2 (0.03 mmol), and C_3 (0.06 mmol), were prepared from the stock linker solution.

To create the concentrations C1, C2, and C3, 25 µL, 75 µL, and 150 µL of the stock linker solution were transferred into 2 mL centrifuge tubes, respectively. Subsequently, 225 µL, 175 µL, and 100 µL of 10 % NaOD/D_2O and 250 µL of the internal standard reference NaO_2CH were added to each sample.

For the analysis of CAU-52, 5.5 mg of the compound was weighed into centrifuge tubes, followed by the addition of 250 µL of 10 % NaOD/D_2O and 250 µL of the internal standard NaO_2CH. The samples were then shaken for 15 minutes and centrifuged for 5 minutes.

The quantification of the linker content in CAU-52 was performed by using the ratio of the characteristic integrals of the linker and the internal reference signal (NaO_2CH) from the ^{1}H NMR spectra. This ratio corresponds to the linker amounts in C1, C2, and C3 (Fig. S10 and Table S6).

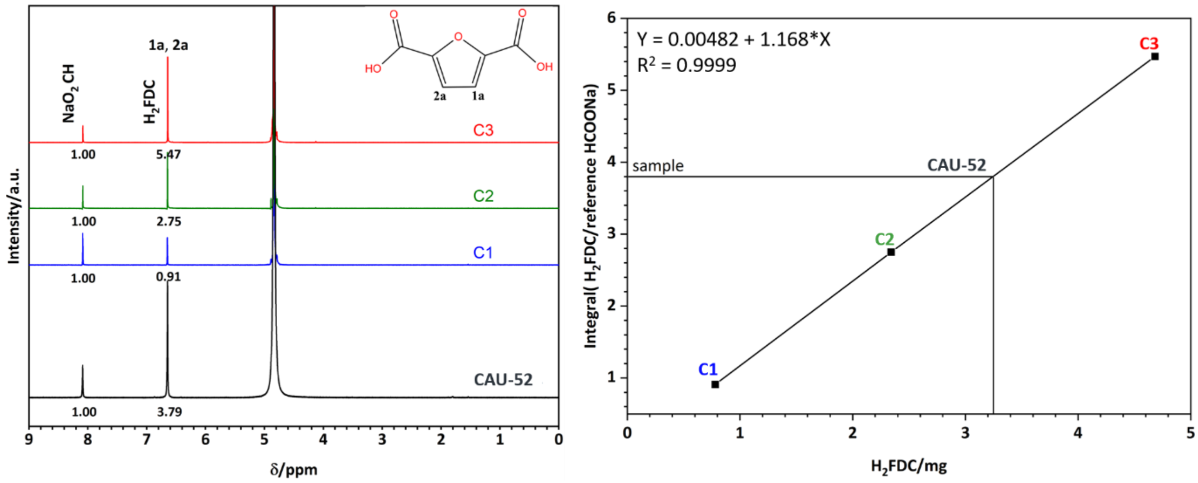

Fig. S10. ^{1}H NMR spectra showing the characteristic peaks of the linker and internal standard at 6.62 and 8.08 ppm (left). Correlation between the amount of linker and the ratio of the observed integrals of the linker to internal standard (right). The amount of linker molecule in CAU-52 was calculated from the ratio of the observed integrals (3.79).

Table S6. Details of the quantification of linker molecules in CAU-52. C1, C2 and C3 are the samples with different amounts of H_2FDC and a constant amount of NaO_2CH used to generate the calibration curve. The integral ratio of 3.79 corresponds to 3.24 mg of linker in CAU-52 confirming the composition $[Fe_3(\mu_3\text{-}O)(FDC)_3(OH)(H_2O)_2]\cdot 5H_2O\cdot H_2FDC$.

**sample**	**H_2FDC amount**		**Integral ratio (H_2FDC / NaO_2CH)**
	**mmol**	**mg**	
C1	0.01	0.78	0.91
C2	0.03	2.34	2.75
C3	0.06	4.68	5.47
CAU-52	-	3.24	3.79

The quantitative analysis shows that sample CAU-52 contains 3.24 mg of the linker in 5.5 mg total mass, corresponding to approximately 58.9% of the total mass. This result is in line with the results of the TG measurements with the second weight loss of 61.39%, thereby confirming the presence of free linker in the pores and the composition $[Fe_3(\mu_3\text{-}O)(FDC)_3(OH)(H_2O)_2]\cdot 5H_2O\cdot H_2FDC$.

3.4. TG analysis

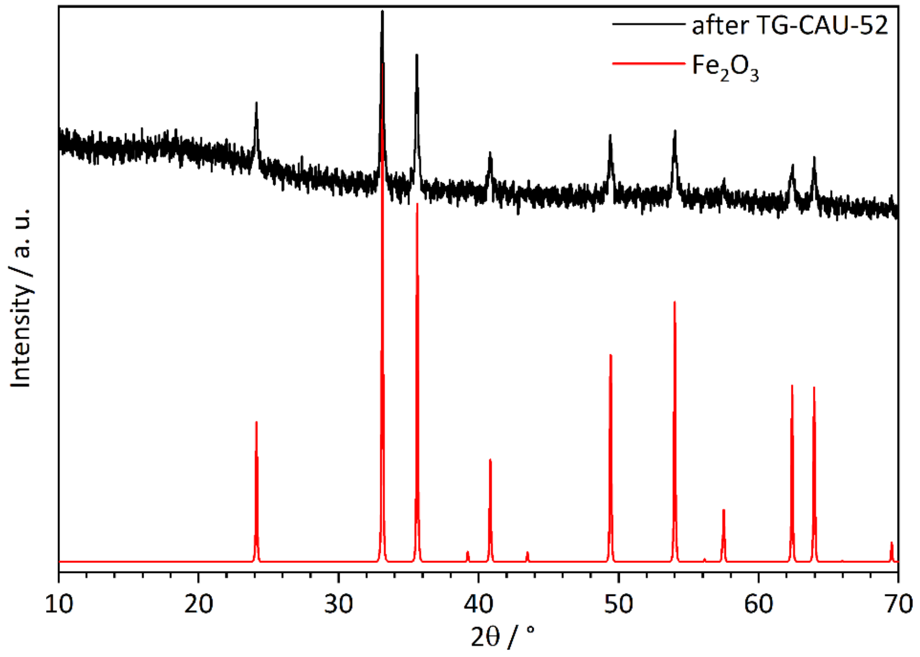

Fig. S11. PXRD pattern of the residue from the TG analysis of CAU-52 (black), compared to a calculated PXRD pattern of Fe_2O_3(red).[5]

The thermogravimetric (TG) measurement of a sample treated for 3 d at 85 % relative humidity was conducted using a Linseis STA PT 1600 instrument (air, flow rate = 6 dm^3/h, heating rate = 8 K/min). The TG curve is shown in Fig. S12 and the quantitative evaluation is presented in Table S7.

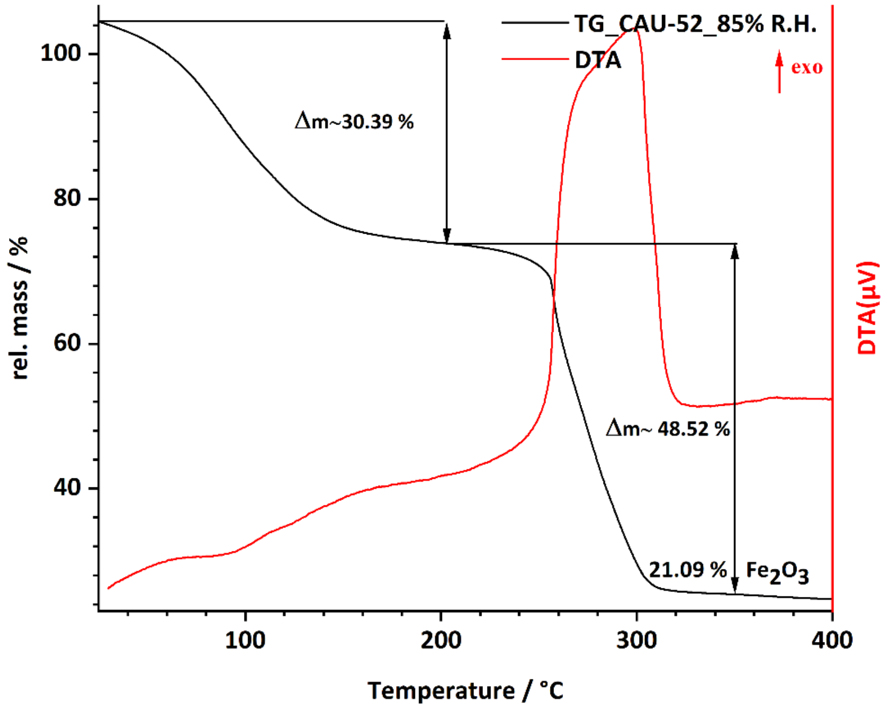

Fig. S12.TG- and DTA curve of CAU-52_85% R.H. Prior to the measurement the sample was treated for 3 d at 85 % relative humidity to assure maximum water adsorption.

Table S7: Quantitative evaluation of the TG curve of CAU-52_85%_R.H. For the calculations the formula $[Fe_3(\mu_3\text{-}O)(FDC)_3(OH)(H_2O)_2]\cdot 18H_2O\cdot H_2FDC$ was used.

Temperature range / °C		Mass loss wt% (obs./calc.)
25 - 200	desorption of 20 water molecules per formular unit	30.39 / 30.56
200 - 350	oxidation of the organic linker and formation of Fe_2O_3	48.52 / 49.13

3.5. Sorption studies

Sorption measurements employing CAU-52 were carried out using H_2O and N_2 as the adsorptives at 298 and 77 K, respectively. PXRD patterns of the samples were recorded before and after the sorption measurements (Fig. S13).

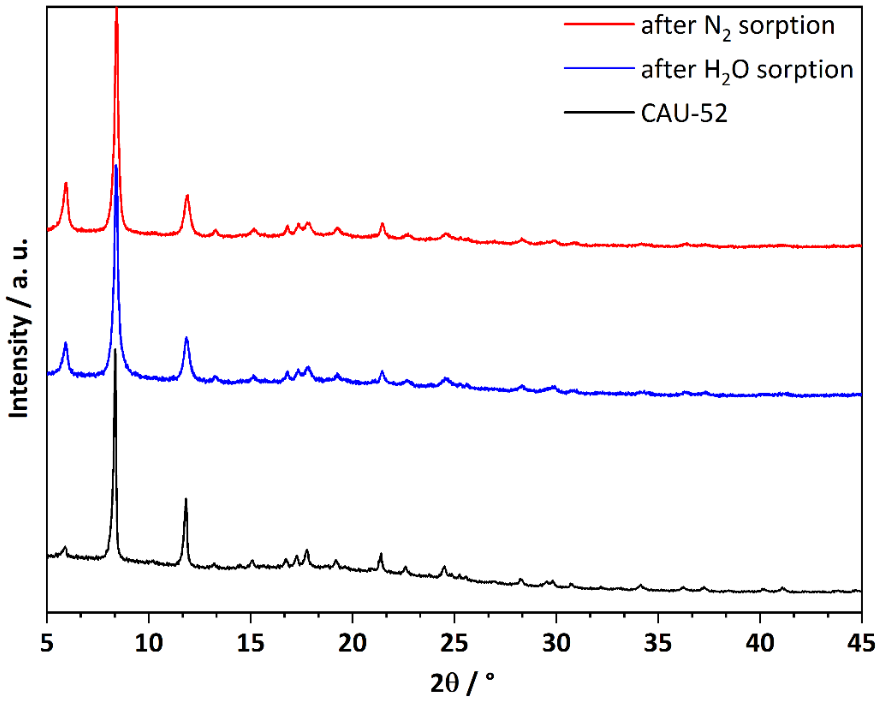

Fig. S13. PXRD patterns of CAU-52 before (blue) and after sorption measurements using H_2O (black) and N_2 (red) as the adsorptive at 298 and 77 K, respectively. Samples after the sorption measurements show a decrease in long range order and differences in the relative intensities, which could be due to the amount residual water molecules in the pores.

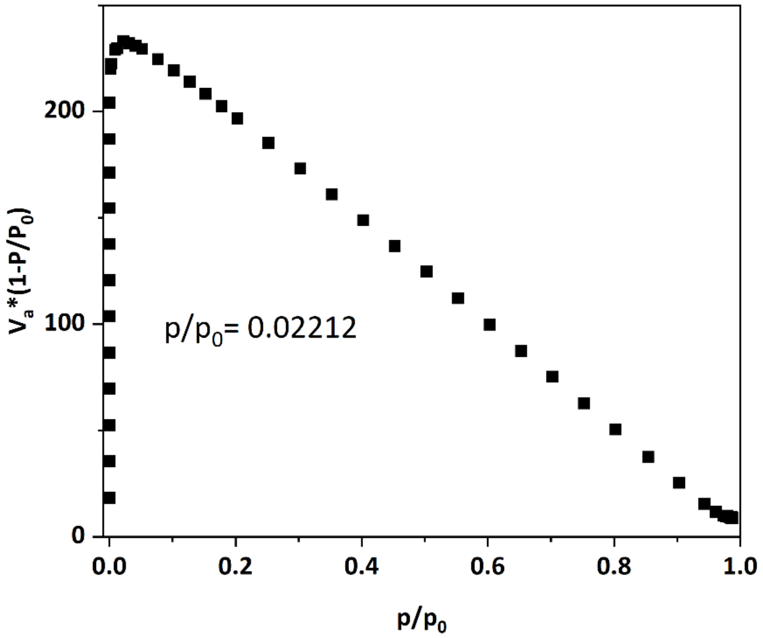

Fig. S14. Rouquerol plot of CAU-52 using the N_2 sorption isotherm collected at 77 K. The value of p/p_0 = 0.02212 was used to determine the maximum relative pressure range for the evaluation using the BET theory.

Repetition of water sorption measurements

To assess the reproducibility of the water sorption capacity of the compound, three consecutive sorption measurements were performed. Prior to the first measurement, the sample was activated at 100 °C for 16 hours. For the subsequent two measurements, a reduced activation time of 3 hours at 100 °C was applied. The sorption isotherms reveal a decreasing water uptake over the course of the repetitions: in the initial measurement, 18.73 mol H_2O per formula unit was adsorbed, which decreased to 17.2 mol H_2O in the first repeated measurement and further declined to 16.0 mol H_2O per formula unit in the second repeated measurement at p/p_0 = 0.86.

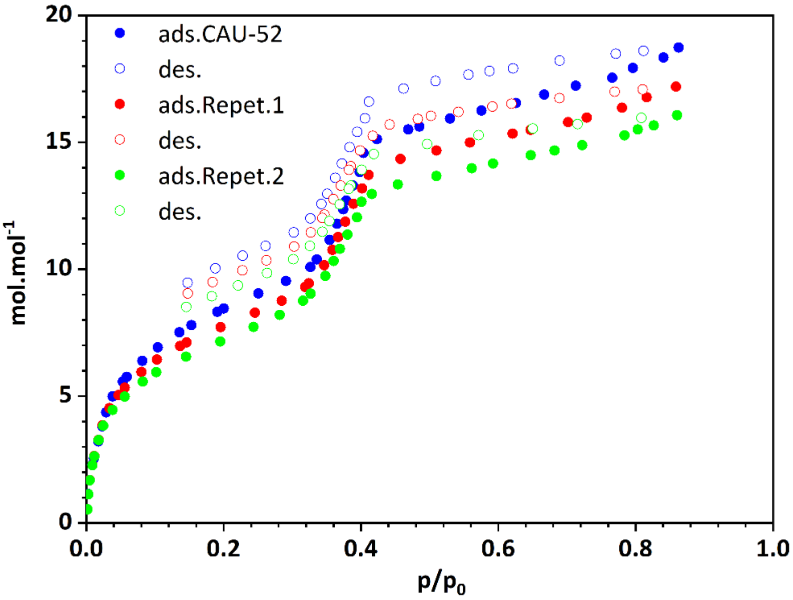

Fig. S15. Three consecutive water sorption isotherms of CAU-52 measured at 298 K. Filled symbols represent adsorption and empty symbols desorption.

4. References

(1) Coelho, A. A. TOPAS and TOPAS-Academic: An optimization program integrating computer algebra and crystallographic objects written in C++. *J. Appl. Crystallogr.* **2018**, *51*, 210–218.

(2) Moghadam, P. Z.; Li, A.; Wiggin, S. B.; Tao, A.; Maloney, A. G. P.; Wood, P. A.; Ward, S. C.; Fairen-Jimenez, D. Development of a Cambridge Structural Database Subset: A Collection of Metal–Organic Frameworks for Past, Present, and Future. *Chem. Mater.* **2017**, *29*, 2618–2625.

(3) Bruno, I. J.; Cole, J. C.; Edgington, P. R.; Kessler, M.; Macrae, C. F.; McCabe, P.; Pearson, J.; Taylor, R. New software for searching the Cambridge Structural Database and visualizing crystal structures. *Acta Cryst. B* **2002**, *58*, 389–397.

(4) Lewis Greenspan. Humidity fixed points of binary saturated aqueous solutions. *JOURNAL OF RESEARCH of the National Bureau of Standards* **1977**, 81A, 89–96.

(5) Gualtieri, A. F.; Venturelli, P. In situ study of the goethite-hematite phase transformation by real time synchrotron powder diffraction. *American Mineralogist* **1999**, *84*, 895–904.
